# Study on the genetic variability and adaptability of turmeric (*Curcuma longa* L.) genotypes for development of desirable cultivars

**DOI:** 10.1371/journal.pone.0297202

**Published:** 2024-01-19

**Authors:** Md. Ashraful Alam, Srabanti Roy, Md. Atikur Rahman, Md. Riazul Islam, Md. Mushfiqur Rahman, Abu Jafor Obaidullah, Md. Nasirul Farid, Md. Marufur Rahman, Md. Rafiqul Islam, Shailendra Nath Mozumder, Riyadh S. Almalki, Ahmed Gaber, Akbar Hossain

**Affiliations:** 1 Plant Breeding Division, Spices Research Centre, Bangladesh Agricultural Research Institute, Bogura, Bangladesh; 2 Department of Agricultural Chemistry, Bangladesh Agricultural University, Mymensingh, Bangladesh; 3 Division of Soil Science, Spices Research Centre, Bangladesh Agricultural Research Institute, Bogura, Bangladesh; 4 Plant Pathology Division, Regional Spices Research Centre, Bangladesh Agricultural Research Institute, Magura, Bangladesh; 5 Division of Horticulture, Spices Research Sub-Centre, Bangladesh Agricultural Research Institute, Faridpur, Bangladesh; 6 Division of Horticulture, Regional Spices Research Centre, Bangladesh Agricultural Research Institute, Magura, Bangladesh; 7 Division of Horticulture, Spices Research Sub-Centre, Bangladesh Agricultural Research Institute, Lalmonirhat, Bangladesh; 8 Regional Station, Bangladesh Institute of Research and Training on Applied Nutrition, Pirganj, Rangpur, Bangladesh; 9 Division of Agronomy, Regional Agricultural Research Station, Bangladesh Agricultural Research Institute, Ishwardi, Pabna, Bangladesh; 10 Division of Horticulture, Spices Research Centre, Bangladesh Agricultural Research Institute, Bogura, Bangladesh; 11 Department of Pharmacology and Toxicology, Faculty of Pharmacy, Umm AL-Qura University, Mecca, Saudi Arabia; 12 Department of Biology, College of Science, Taif University, Taif, Saudi Arabia; 13 Division of Soil Science, Bangladesh Wheat and Maize Research Institute, Dinajpur, Bangladesh; KGUT: Graduate University of Advanced Technology, ISLAMIC REPUBLIC OF IRAN

## Abstract

Turmeric, a globally cultivated spice, holds significance in medicine, and cosmetics, and is also a very popular ingredient in South Asian cuisine. A study involving 53 turmeric genotypes evaluated for rhizome yield and related traits at Spices Research Center, Bogura, Bangladesh over three years (2019–22). A randomized complete block design was followed with two replications. ANOVA revealed significant trait variations among genotypes. Genotype T0015 emerged as the highest yielder at 28.04 t/ha. High heritability (0.58–0.99) and genetic advance characterized plant height (PH), mother rhizome weight (WMR), primary and secondary finger weights (WPF and WSF), and yield per plant (YPP) across seasons. Genetic gain (GG) was prominent in these traits. Genotypic and phenotypic coefficient variations (GCV and PCV) (6.24–89.46 and 8.18–90.88, respectively) across three years highlighted mother rhizome weight’s importance followed by numbers of primary finger (NPF), and WPF. Positive and significant correlations, especially with PH, WMR, WPF, and YPP, emphasized their relevance to fresh yield (FY). Multiple linear regression identified PH, number of mother rhizome (NMR) and WMR as key contributors, explaining 37–79% of FY variability. Cluster analysis grouped genotypes into five clusters with maximum distance observed between clusters II and III. The geometric adaptability index (GAI) assessed adaptability and superiority, revealing nine genotypes outperforming the best existing cultivar. Genotype T0117 as the top performer based on GAI, followed by T0103 and T0094. Mean rank analysis favoured T0121 as the best performer, succeeded by T0117, T0082 and T0106. The top ten genotypes (T0015, T0061, T0082, T0085, T0094, T0103, T0106, T0117, T0121 and T0129) were identified as superior based on yield and overall ranking, warranting further evaluation. These findings may induce a window for improving turmeric research and ultimately play a role in enhancing its cultivation and productivity.

## 1. Introduction

Turmeric (*Curcuma longa* L.) is a cross-pollinated, triploid (2n = 3x = 63) rhizome crop grown worldwide [1]. It ranks among the most popular crops globally, boasting a pungent favour and an attractive colour. Turmeric serves various purposes, such as a spice, cosmetic, colorings agent, favouring and preservative, and is recognized universally for its aromatic, stimulative, and carminative properties. It is marketed in various forms, including spice, dye, oleoresin, complexion agent, and industrial starch source [2–4]. Additionally, turmeric is a key ingredient in many South Asian cuisines, and it is sometimes consumed as tea or in pill form. It finds application in numerous industrial products, including sauces, mustards, dry seasonings, baking mixes, processed cheese, dry soups, and confections [1]. Ayurvedic medicine also values turmeric, particularly for its bioactive element, curcumin [5]. Curcuminoids, which are fat-soluble, polyphenolic pigments, impart a bright yellow colour to turmeric. Furthermore, turmeric is employed in formulating human medicines due to its anti-inflammatory and antiseptic properties [6], as well as its ability to inhibit carcinogenesis [7, 8].

Globally, around 11 lakh metric tons of turmeric are produced annually, with India leading in production, consumption, and export. India accounts for 80% of the world’s turmeric production, followed by China (8%), Myanmar (4%), Nigeria (3%), and Bangladesh (3%). Bangladesh, for instance, imports approximately 49,522 metric tons of turmeric from India each year [9].

Turmeric primarily propagates through its underground rhizomes [1], with seed production being rare. Hybridization is generally unproductive, making genetic improvement limited to germplasm selection and mutation breeding [8]. Chandra et al. [10] emphasized the importance of germplasm collection for turmeric genetic improvement, contributing significantly to genetic diversity and variability. It is the basic indispensable element of all breeding programs. Exploiting the existing variability in germplasm collections is crucial for genotype selection in breeding programs [11].

Various statistical tools are pivotal in turmeric evaluation, offering insights for superior genotype selection and managing germplasm variability. Techniques like variability estimates provide a comprehensive understanding of turmeric diversity, aiding strategic genotype selection [12–14]. Whereas, clustering analysis categorizes genotypes based on shared characteristics, revealing patterns [14–16]. Similarly, trait association analysis uncovers trait relationships, guiding the selection of favourable combinations [17, 18]. On the other hand, genotype stability and adaptability analysis assess performance across environments, ensuring stable and adaptable genotypes are selected, collectively enhancing the success of turmeric breeding and germplasm improvement [4]. These methods enable effective utilization of germplasm variability, ensuring precise and comprehensive evaluations for identifying promising turmeric cultivars.

In the realm of turmeric evaluation, various statistical tools and techniques play a crucial role in extracting meaningful insights and guiding the selection of superior genotypes. These tools are instrumental in handling the inherent variability within germplasm collections, ensuring a comprehensive understanding of the performance of different turmeric cultivars.

Despite turmeric being cultivated throughout Bangladesh, both its area and production are declining despite increasing yield potential [19]. The country produced 2.18 million metric tons of turmeric from 0.26 lakh ha of land during 2020–21, with an average yield of 8.24 t/ha [19]. This yield is considerably lower compared to other countries. It is a significant source of income for the hilly regions of Bangladesh. Despite the country’s production, it falls short of demand, leading to imports to meet local needs [20]. As demand rises and cultivation area diminishes, the introduction of high-yielding varieties could contribute to increased production. The Spices Research Center (SRC), Bangladesh’s sole public research center for turmeric improvement, has developed five improved varieties to date. However, more robust genotypes are required to meet demands in terms of yield, quality, and tolerance to diseases and environmental stresses (salinity, drought, etc.). Recent research indicates that replacing traditional cultivars with agro-ecologically adaptable ones can significantly boost yields [21]. With these considerations in mind, this study aims to evaluate the performance of promising turmeric genotypes across seasons and identify potential lines for progeny evaluations in METs.

## 2. Materials and methods

### 2.1. Genotypes

A total of 53 turmeric genotypes collected from all over Bangladesh were evaluated during three consecutive seasons. Five commercially available turmeric varieties developed by a public research institute were used as standard checks. Details of the genotypes were given in S1 Table.

### 2.2. Location

From April to February during three consecutive seasons of 2019–20, 2020–21 and 2021–22, those turmeric lines were evaluated at the Spices Research Centre, BARI, Shibganj, Bogura (24°58’35” North latitude and 89°20’11” East longitude), which belongs to the AEZ-4 (FAO, 1988). The soils were characterized by grey silt loams and silty clay loams on ridges and grey or dark grey clays in basins, while being moderately acidic in nature with low organic matter content. The site of the study was covered by a sub-tropical climate, with hot and dry weather prevailing during March to May and cool and dry weather during November to February. The temperature during the monsoon (June to September) was moderately hot but highly humid with a mean annual rainfall of 1520 mm to 1990 mm. The weather details that prevailed during the cropping seasons of different years at the location were given in S2 Table. The physical properties of the initial soil of the experimental site were presented in S3 Table and the chemical properties of the initial and post-harvest soil of the experimental site were provided in S4 Table.

### 2.3. Experimental details

The study was one of the national and regular experiments of the Spices Research Centre of Bangladesh Agricultural Research Institute (BARI), Shibganj, Bogura-Bangladesh for the development of crop cultivars suitable for the condition of Bangladesh. The research was conducted with the permission and financial support of the director general of Bangladesh Agricultural Research Institute (BARI).

The field trial was laid out in RCB design to accommodate the genotypes under study with two replications. The seed rhizomes of the different genotypes were planted on well-prepared raised beds on 17 April 2019, 13 April 2020, and 23 April 2021 during three consecutive seasons, respectively. The unit plot was 3m x 1.8m in size, with 0.5m space between beds. A 60 cm x 25 cm spacing was maintained from row to row and hill to hill during the planting of seed rhizomes. The experimental field received 5 t/ha of cow dung one week before sowing, while chemical fertilizers N, P, K, S, and Zn (at 120, 36, 85, 20, and 2 kg/ha) were applied immediately before sowing, except for N, which was applied in three splits. Other intercultural practices such as weeding were done at the vegetative stage and earthing up was done twice during the crop cycle. Timely irrigation was provided to ensure moisture availability and plant protection measures in the form of scheduled spraying were done to repel pest infestation.

### 2.4. Data collection

Using the standard protocol for turmeric phenotyping [8, 15], observations of different morpho-physiological traits were recorded. Plant height, number of branches, and number of leaves were measured and averaged on randomly selected plants during the second week of November. At maturity, the number and weight of the mother rhizome, primary and secondary fingers, length of the mother rhizome, and length and breadth of fingers were recorded based on randomly taking five plants and averaging them. The fresh yield was recorded at harvest on a whole plot basis and converted to tons per hectare.

### 2.5. Statistical analysis

The analysis of variance for individual traits was carried out using R software [16] using ’Agricolae’ package [17]. Analysis was required to test whether the genotypes differed significantly among themselves or not. Phenotypic and genotypic variance and heritability in the broad sense for all the traits were estimated according to the formula as suggested by Lush [18] and were classified into low (0–30%), moderate (30–60%) and high (>60%) as suggested by Robinson et al. [19]. Analysis of genotypic and phenotypic associations was performed in ‘META-R’ software [20] on all seasons data to observe the association among studied traits. Multiple linear regression was performed to visualize the extent of the relationship between different important traits and final fresh yield using the ‘ggplot2’ package [21] in ‘R’ software [16]. The clustering of the studied genotypes based on trait performances was done through cluster analysis suggested by D2 analysis [22, 23] done in the ‘R’ platform [16]. The Geometric Adaptability Index (GAI), which is a measure of the adaptability of a genotype across different environments proposed by Mohammadi and Amri [24] is considered a dynamic concept of stability which is being used in different studies [25–27]. GAI was estimated using the ‘metan’ package [28] in ‘R’ platform [16].

## 3. Results

### 3.1 Variability in growth and yield traits

This particular study was executed to explore the variability present for different yields and its attributing traits in fifty-three turmeric genotypes. The observations recorded on twelve traits were evaluated in three seasons and analyzed statistically. The analysis of variance of the studied traits of turmeric is presented in Table 1. The variance due to genotypes was significant (p≤0.05) for the traits across years. The present study during three years disclosed a wide range of variations for the traits under consideration.

**Table 1:** Estimation of genetic parameters in twelve traits of 53 genotypes of turmeric grown over the years.

Traits	Max	Min	X	SE	CV%	(CD) 5%	(CD) 1%	σ^2^e	σ^2^g	σ^2^p	ECV	GCV	PCV	h^2^_b_	GA	GG
**2019–2020**																
PH	140.36	54.13	86.94	6.03	9.81	17.11	22.79	72.67	275.09	347.76	9.81	19.08	21.45	0.79	30.39	34.95
NB	4.49	1.34	2.52	0.17	9.41	0.48	0.64	0.06	0.34	0.39	9.43	23.03	24.88	0.86	1.11	43.90
NL	31.24	10.67	18.62	1.32	9.99	3.73	4.97	3.46	22.93	26.39	9.99	25.73	27.60	0.87	9.20	49.40
NMR	2.75	0.92	1.61	0.11	9.66	0.31	0.42	0.02	0.20	0.22	9.66	27.52	29.17	0.89	0.86	53.48
WMR	188.38	9.46	58.91	5.21	12.52	14.80	19.72	54.39	1094.78	1149.16	12.52	56.17	57.55	0.95	66.53	112.94
NPF	10.76	1.94	5.67	0.41	10.15	1.15	1.54	0.33	3.58	3.92	10.15	33.42	34.93	0.92	3.73	65.87
WPF	173.80	17.28	64.27	5.50	12.11	15.62	20.81	60.57	1188.63	1249.20	12.11	53.64	54.99	0.95	69.28	107.79
NSF	16.78	2.91	8.38	0.61	10.28	1.73	2.30	0.74	9.67	10.42	10.28	37.13	38.52	0.93	6.18	73.71
WSF	118.80	8.97	38.00	3.27	12.15	9.27	12.35	21.32	583.57	604.89	12.15	63.57	64.72	0.96	48.88	128.63
LMR	10.07	3.49	6.04	0.42	9.74	1.18	1.57	0.35	1.30	1.65	9.74	18.91	21.27	0.79	2.09	34.62
YPP	1603.80	47.53	242.51	27.40	15.98	77.75	103.60	1501.28	47066.27	48567.55	15.98	89.46	90.88	0.97	439.95	181.42
FY	30.80	1.46	10.05	0.99	13.91	2.80	3.74	1.95	49.29	51.24	13.91	69.88	71.25	0.96	14.18	141.17
**2020–2021**																
PH	118.98	57.47	100.24	3.75	5.29	10.64	14.18	28.14	39.15	67.29	5.29	6.24	8.18	0.58	9.83	9.81
NB	4.12	2.05	2.91	0.11	5.27	0.31	0.41	0.02	0.09	0.11	5.31	10.28	11.57	0.79	0.55	18.82
NL	34.86	10.10	25.72	0.98	5.39	2.78	3.71	1.92	12.61	14.53	5.39	13.81	14.82	0.87	6.81	26.50
NMR	3.09	0.95	1.51	0.06	5.66	0.17	0.23	0.01	0.22	0.22	5.65	30.73	31.25	0.97	0.94	62.26
WMR	314.18	33.95	127.40	4.96	5.50	14.07	18.75	49.18	3592.61	3641.79	5.50	47.05	47.37	0.99	122.64	96.26
NPF	9.19	0.97	2.15	0.10	6.74	0.29	0.39	0.02	1.07	1.09	6.71	48.23	48.70	0.98	2.11	98.42
WPF	247.16	42.40	97.92	3.94	5.69	11.17	14.89	31.01	1751.90	1782.90	5.69	42.75	43.12	0.98	85.47	87.29
NSF	22.31	5.83	11.69	0.46	5.61	1.32	1.76	0.43	9.36	9.80	5.62	26.17	26.77	0.96	6.16	52.71
WSF	529.20	22.09	205.88	8.67	5.96	24.61	32.79	150.38	7732.94	7883.32	5.96	42.71	43.13	0.98	179.41	87.15
LMR	12.76	5.06	8.98	0.33	5.27	0.95	1.26	0.22	1.47	1.69	5.27	13.50	14.49	0.87	2.33	25.90
YPP	1161.83	146.60	488.05	20.04	5.81	56.88	75.79	803.48	36257.85	37061.33	5.81	39.02	39.45	0.98	387.98	79.50
FY	36.55	4.19	20.96	0.81	5.47	2.30	3.06	1.31	57.99	59.31	5.46	36.34	36.75	0.98	15.51	74.03
**2021–2022**																
PH	139.53	77.19	106.16	5.40	7.19	15.31	20.40	58.23	136.59	194.82	7.19	11.01	13.15	0.70	20.16	18.99
NB	11.56	2.47	5.62	0.30	7.65	0.86	1.15	0.18	2.48	2.67	7.65	28.01	29.04	0.93	3.13	55.67
NL	39.16	13.02	24.83	1.29	7.36	3.66	4.88	3.33	24.08	27.41	7.35	19.77	21.09	0.88	9.48	38.16
NMR	3.41	0.93	1.44	0.07	7.38	0.21	0.28	0.01	0.23	0.25	7.35	33.67	34.46	0.95	0.97	67.76
WMR	315.00	23.04	77.47	4.39	8.01	12.45	16.59	38.51	1977.05	2015.56	8.01	57.40	57.95	0.98	90.72	117.10
NPF	16.01	1.19	6.35	0.32	7.02	0.90	1.19	0.20	4.76	4.96	7.02	34.35	35.06	0.96	4.40	69.33
WPF	319.99	40.61	112.88	6.50	8.15	18.45	24.59	84.55	2741.62	2826.17	8.15	46.39	47.10	0.97	106.24	94.12
NSF	16.01	3.49	7.98	0.42	7.48	1.20	1.60	0.36	6.35	6.71	7.48	31.59	32.46	0.95	5.05	63.32
WSF	384.30	27.89	87.81	5.39	8.68	15.29	20.37	58.07	3076.71	3134.78	8.68	63.17	63.76	0.98	113.20	128.92
LMR	9.92	5.19	7.35	0.38	7.29	1.08	1.43	0.29	0.86	1.15	7.30	12.63	14.59	0.75	1.66	22.54
YPP	721.09	97.27	281.32	15.71	7.90	44.57	59.39	493.43	15418.26	15911.70	7.90	44.14	44.84	0.97	251.79	89.51
FY	47.70	3.80	19.54	1.09	7.89	3.09	4.12	2.38	90.37	92.74	7.89	48.65	49.29	0.97	19.33	98.93

PH = Plant Height; NB = Number of branches; NL = Number of leaves; NMR = Number of mother rhizome; WMR = Weight of mother rhizome; NPF = Number of primary fingers; WPF = Weight of primary finger; NSF = Number of secondary fingers; WSF = Weight of secondary finger; MRL = Length of mother rhizome; YPP = Yield per plant; FY = Fresh yield; Max = Maximum; Min = Minimum; X = Mean value; SE = Standard error; CV% = Coefficient of variation; CD = Critical difference at 1% and 5% level of significance; σ^2^e = Environmental variance; σ^2^g = Genotypic variance; σ^2^p = Phenotypic variance; ECV = Environmental coefficient of variation; GCV = Genotypic coefficient of variation; PCV = Phenotypic coefficient of variation; h^2^_b_ = Heritability; GA = Genetic advance; GG = Genetic gain.

During the seasons 2019–20; 2020–21 and 2021–22, coefficients of variation ranged from 9.41 to 15.98; 5.27 to 6.74 and 7.02 to 8.68 percent, respectively. The higher genotypic coefficient of variation (GCV) was recorded for the traits WMR (56.17), WPF (53.64), WSF (63.57), YPP (89.46) and FY (69.88) in the first year (Table 1); WMR (47.05), NPF (48.23), WPF (42.75), WSF (42.71), YPP (39.02), FY (36.34) in the second year; and WMR (57.40), WPF (46.39), WSF (63.17), YPP (44.14) and FY (48.65) in the third year. Contrary to this, other traits showed a moderate to low genotypic coefficient of variation.

On the other hand, the higher phenotypic coefficient of variation (PCV) was recorded for the traits WMR (57.77), WPF (54.99), WSF (64.72), YPP (90.88) and FY (71.25) in the first year (Table 1); WMR (47.37), NPF (48.70), WPF (43.12), WSF (43.13), YPP (39.45), FY (36.75) in the second year; and WMR (57.95), WPF (47.10), WSF (63.76), YPP (44.84) and FY (49.29) in the third year. In contrast, the rest of the traits’ phenotypic coefficient of variation were moderate to low.

### 3.2 Heritability, genetic advance and genetic gain of different traits

Heritability for different traits varied from 88 to 98; 74 to 99 and 82 to 99 percent during the seasons of 2019–20; 2020–21 and 2021–22, respectively. In the year 2019–20, high estimates of heritability (>0.80) were observed for the number of branches (0.86), number of leaves (0.87), number of mother rhizomes (0.89), number of primary fingers (0.92), weight of primary fingers (0.95), yield per plant (0.97) and fresh yield (0.96). In the second year 2020–21, leaves (0.87), mother rhizomes (0.97), weight of mother rhizomes (0.99), primary fingers (0.98), weight of primary fingers (0.98), secondary fingers (0.96), weight of secondary fingers (0.98), length of mother rhizomes (0.87), yield/plant (0.98) and fresh yield (0.98) showed a high level of heritability. Whereas, during the year 2021–22, branches (0.93), leaves (0.88), mother rhizomes (0.95), weight of mother rhizomes (0.98), primary fingers (0.96), weight of primary fingers (0.97), secondary fingers (0.98), weight of secondary fingers (0.98), yield/plant (0.97) and fresh yield (0.97) showed high heritability.

A wider range of GA was estimated for different traits during all the years (0.86–439.95 in 2019–20; 0.55–387.98 in 2020–21; and 0.97–251.79 in 2021–22) (Table 1). The higher GA was observed for the traits such as plant height (30.39), WMR (66.53), WPF (69.28), WSF (48.88), and yield per plant (439.95) in the first year; weight of mother rhizome (122.64), WPF (85.47), weight of secondary finger (179.41), and YPP (387.98) in the second year; and WMR (90.72), WPF (106.24), WSF (113.20), and YPP (251.79) in the year III.

A broader range of GG was also found for different traits during all the years (34.62–181.42 in 2019–20; 9.81–98.24 in 2020–21; and 18.99–128.92 in 2021–22) (Table 1). The higher genetic gain (GG) was observed for the trait WMR (112.94), weight of primary finger (107.79), WSF (128.63), YPP (181.242) and fresh yield (141.17) while the minimum was obtained in the first year; the higher GG during year II was for WMR (96.26) and number of primary finger (98.42) and the minimum gain obtained in PH (9.81); whereas higher GG was observed for weight of mother rhizome (117.10) and weight of secondary finger (128.92) in the third year, in which the minimum was estimated for PH (18.99).

### 3.2 Genotypes performance for different traits

#### 3.2.1 Combined performance over the years

The evaluation of various turmeric genotypes over three seasons reveals distinct characteristics across multiple traits (S5 Table). Notably, genotype T0117 emerges as the tallest variety at 116.50 cm, closely followed by the genotypes T0129, T0103, and T0121. In contrast, genotype T0127 stands out as the most dwarf variety at 71.13 cm, with genotypes T0122, T0128, and T0054 as subsequent dwarf varieties. Moving to rhizome traits, genotype T0121 leads with 2.25 mother rhizomes, weighing 142.96 g, while genotype T0094 closely follows with 2.17 and a rhizome weight of 111.67 g. Conversely, T0093 and T0108 exhibit the minimum number of mother rhizomes (1.08 each) with weights of 82.83 g and 35.68 g, respectively. Genotype T0017, with a solitary mother rhizome, weighs 65.67g. Examining primary fingers, genotype T0098 boasts the maximum count at 6.25, with a weight of 94.03g, while T0019 follows closely with 6.17 and a weight of 91.97g. Genotypes T0052 and T0109 display the minimum primary finger count (2.50 each) and weigh 48.83g and 38.27g, respectively. In secondary fingers, genotype T0082 leads with 13.67, weighing 145.92g, followed by T0126 with 12.25 and 197.28g. Genotype T0052, with 5.17 secondary fingers, weighs 71.75g, and T0109, with 5.59, weighs 56.04g. Genotype T0103 claims the highest number of branches at 5.80 and 30.81 leaves, while genotype T0023 with the minimum leaf count at 2.40 and 23.73 leaves. Genotype T0093 exhibits the maximum mother rhizome length at 9.03, and T0077 boasts the longest mother at 5.28. In terms of yield, genotype T0123 records the maximum at 743.55g, while genotype T0109 notes the minimum at 120.46g. Genotype T0015 excels with the highest fresh yield at 28.04, equivalent to 269.92g per plant. The average fresh yield ranges between 17.74∼18.0, and genotype T0127, with the lowest fresh yield at 4.00, may be omitted if prioritizing fresh yield in breeding, with genotype T0015 being a favoured selection.

#### 3.2.2 Year 2019–20

During the 2019–20 evaluation, the tallest plant was recorded in T0103 (127.60 cm) while the dwarf one was recorded in T0077 (57.20 cm) (S6 Table). The highest number of tillers (4.20) was obtained from T0093, and the lowest number of tillers (1.40) was recorded in T0052. The maximum number of leaves (29.40) was found in T0084, and the minimum number of leaves (11.00) was found in T0077. The maximum number of mother rhizomes (2.51) was recorded in T0134 and the minimum number (1.00) was obtained from T0063. The heavier mother rhizome (171.25 g) was recorded from T0085, while the lowest weight of the mother rhizome (9.75 g) was observed from T0122. The maximum number of primary fingers (10.25) was recorded in T0096, and the minimum number of primary fingers (2.00) was noted in T0122. The heavier primary fingers (158.00 g) were recorded in T0085 while the lowest (18.00 g) were recorded in T0127. The maximum number of secondary fingers (15.26) was found in T0082 while the minimum number of secondary fingers (3.00) was recorded in T0104. The heavier secondary finger (108.00 g) was found in T0121, while the lowest (9.25 g) was recorded in T0122. The maximum mother rhizome length (9.15 cm) was documented from BARI Holud-4. The highest yield per plant (1458.00g) was obtained from T0121, and the lowest yield per plant (49.00g) was attained from T0109. The highest fresh yield (28.00 t/ha) was observed from T0106, and it was followed by T0117 (26.06 t/ha).

#### 3.2.3 Year 2020–21

In the 2020–21 season trial, the tallest plant was recorded in T0082 (114.40 cm) while the dwarf one was from T0127 (61.80 cm) (S7 Table). The highest number of tillers (4.00) was obtained from T0102 and the lowest number of tillers (2.20) was recorded from T0127. The most number of leaves (33.20) were found in T0017 and the fewest leaves (10.41) were recorded from T0096. The maximum number of mother rhizomes (3.00) was obtained from T0117. The heavier mother rhizomes (305.03 g) were recorded in T0117, while the lowest weight of mother rhizomes (36.51 g) was found in T0127. The maximum number of primary fingers (8.75) was recorded in T0107, and the minimum number of primary fingers (1.00) was documented in T0134. The heavier primary fingers (239.96 g) were recorded in T0103 while the lowest (43.79 g) was noted in T0109. The maximum number of secondary fingers (21.25) was found in T0008 while the minimum number of secondary fingers (6.01) was recorded in T0108. The heavier secondary fingers (504.00 g) were recorded in T0008 while the lowest (23.75 g) were found in T0127. The maximum mother rhizome length (12.51 cm) was recorded in T0124. The highest yield per plant (1106.50 g) was obtained from T0008 and the lowest yield per plant (151.13g) was noted from T0109. The highest fresh yield (35.83 t/ha) was documented from T0123, and it was followed by T0019 (34.76 t/ha).

#### 3.2.4 Year 2021–22

On the other hand, in the 2021–22 experiment, the tallest plant was recorded in T0118 (130.40 cm) while the dwarf one was in T0108 (83.00 cm) (S8 Table). The highest number of tillers (10.80) was obtained in T0103 and the lowest number of tillers (2.60) was recorded in T0023. Maximum leaves (36.60) were found in T0103 and the minimum number of leaves (14.00) was recorded in T0108. The maximum number of mother rhizomes (3.25) was found in T0132. The heavier mother rhizome (300.00 g) was recorded in T0132, while the lowest weight of the mother rhizome (24.25 g) was obtained from T0133. The maximum number of primary fingers (15.25) was recorded in T0132 and the minimum number of primary fingers (1.25) was noted in T0023. The heavier primary finger (304.25 g) was recorded in T0132 while the lowest (42.75 g) was found in T0123. The maximum number of secondary fingers (15.25) was found in T0126 while the minimum number of secondary fingers (3.75) was recorded in T0109. The heavier secondary finger (366.00 g) were documented in T0126 while the lowest (28.50 g) was recorded in T0095. The maximum mother rhizome length (9.45 cm) was recorded in T0132. The highest yield per plant (686.75 g) was obtained from T0132 and the lowest yield per plant (99.26g) was observed from T0095. The highest fresh yield (45.00 t/ha) was obtained from T0015s and it was followed by T0061 (42.00 t/ha).

### 3.3 Association of traits

Association analyses for studied traits were performed at both the phenotypic and genotypic levels in the current study (Table 2 and S9–S11 Tables). In all three years as well as when combined over years, a higher genetic correlation was observed than the phenotypic counterpart in this investigation. Over the years, results of the traits association study revealed that FY had a strong and positive association with all the traits at both genotypic and phenotypic levels except for NMR and NPF at the genotypic level. The maximum value of association for FY was found with PH (0.99^**^ and 0.75^**^) which was closely followed by WMR (0.99^**^ and 0.70^**^), WPF (0.99^**^ and 0.59^**^) and YPP (0.99^**^ and 0.58^**^) (Table 2). Among other morphological traits, a notable positive and significant association was found between WMR and YPP (0.99^**^ and 0.77^**^) which was closely followed by WPF and YPP (0.99^**^ and 0.70^**^), WSF and YPP (0.99^**^ and 0.70^**^) and between WMR and WPF (0.60^**^ and 0.67^**^) LMR and WMR (0.99^**^ and 0.57^**^), WPF and WSF (0.99^**^ and 0.53^**^) etc. (Table 2).

**Table 2:** Association of studied traits based on combined performances over the years.

Traits	Type	PH	NB	NL	NMR	WMR	NPF	WPF	NSF	WSF	LMR	YPP
NB	r_g_	0.99^**^										
	r_p_	0.39^*^										
NL	r_g_	0.68^**^	0.99^**^									
	r_p_	0.58^**^	0.59^**^									
NMR	r_g_	-	-	-								
	r_p_	0.25	0.06	0.20								
WMR	r_g_	0.99^**^	0.99^**^	0.99^**^								
	r_p_	0.70^**^	0.36^**^	0.44^**^	0.46^**^							
NPF	r_g_	-	-	-	-	-						
	r_p_	0.37^**^	0.29^*^	0.40^**^	0.37^**^	0.50^**^						
WPF	r_g_	0.99^**^	0.99^**^	0.99^**^	-	0.60^**^						
	r_p_	0.42^**^	0.41^**^	0.41^**^	0.33^*^	0.67^**^	0.54^**^					
NSF	r_g_	0.63^**^	0.99^**^	0.99^**^	-	0.99^**^	-	0.58^**^				
	r_p_	0.31^*^	0.25	0.47^**^	0.29^*^	0.55^**^	0.47^**^	0.47^**^				
WSF	r_g_	0.85^**^	-0.46^**^	0.22	-	0.38^*^	-	0.99^**^	-0.08			
	r_p_	0.34^*^	-0.01	0.19	0.29^*^	0.52^**^	0.11	0.53^**^	0.52^**^			
LMR	r_g_	0.96^**^	0.99^**^	0.83^**^	-	0.99^**^	-	0.78^**^	0.99^**^	0.15		
	r_p_	0.52^**^	0.27	0.40^**^	-0.12	0.57^**^	0.44^**^	0.37^**^	0.42^**^	0.15		
YPP	r_g_	0.99^**^	0.99^**^	0.99^**^	-	0.99^**^	-	0.99^**^	0.80^**^	0.99^**^	0.99^**^	
	r_p_	0.47^**^	0.24	0.49^**^	0.48^**^	0.77^**^	0.47^**^	0.70^**^	0.61^**^	0.70^**^	0.32^**^	
FY	r_g_	0.99^**^	0.99^**^	0.99^**^	-	0.99^**^	-	0.99^**^	0.64^**^	0.68^**^	0.62^**^	0.99^**^
	r_p_	0.75^**^	0.53^**^	0.55^**^	0.36^**^	0.70^**^	0.49^**^	0.59^**^	0.33^*^	0.34^*^	0.39^**^	0.58^**^

* 5% level of probability

** 1% level of probability; PH = Plant Height; NB = Number of branches; NL = Number of leaves; NMR = Number of mother rhizome; WMR = Weight of mother rhizome; NPF = Number of primary fingers; WPF = Weight of primary finger; NSF = Number of secondary fingers; WSF = Weight of secondary finger; MRL = Length of mother rhizome; YPP = Yield per plant; FY = Fresh yield; r_g_: genotypic correlation coefficient; r_p_: phenotypic correlation coefficient.

In the year 2019–20, the maximum value of positive and significant association for FY was found with PH (0.81^**^ and 0.80^**^) which was closely followed by WMR (0.78^**^ and 0.78^**^) and WPF (0.77^**^ and 0.77^**^) (S9 Table). Apart from FY, the highest positive and significant association among other traits was found between PH and NB (0.82^**^ and 0.83^**^) followed by WMR and WPF (0.78^**^ and 0.79^**^). A significant and positive association was also observed for FY with WMR (0.55^**^ and 0.56^**^) and WPF (0.44^**^ and 0.45^**^) for the year 2020–21. Among the traits other than FY, the highest value of positive and significant association was obtained between NSF and YPP (0.69^**^ and 0.69^**^) which was closely followed by WMR and YPP (0.64^**^ and 0.65^**^) and WMR and WPF (0.63^**^ and 0.63^**^) (S10 Table). During the year 2021–22, positive and significant associations were recorded for FY with PH (0.35^**^ and 0.36^**^) which was closely followed by WMR (0.32^**^ and 0.32^**^) and WPF (0.30^**^ and 0.33^**^) (S11 Table). Among the yield-contributing traits, notable positive and significant associations were found between WPF and YPP (0.82^**^ and 0.81^**^) which was closely followed by WMR and YPP (0.76^**^ and 0.80^**^), WSF and YPP (0.73^**^ and 0.72^**^) and between WMR and WPF (0.65^**^ and 0.71^**^) (Table 2).

### 3.4 Regression estimates

**Fig 1:**
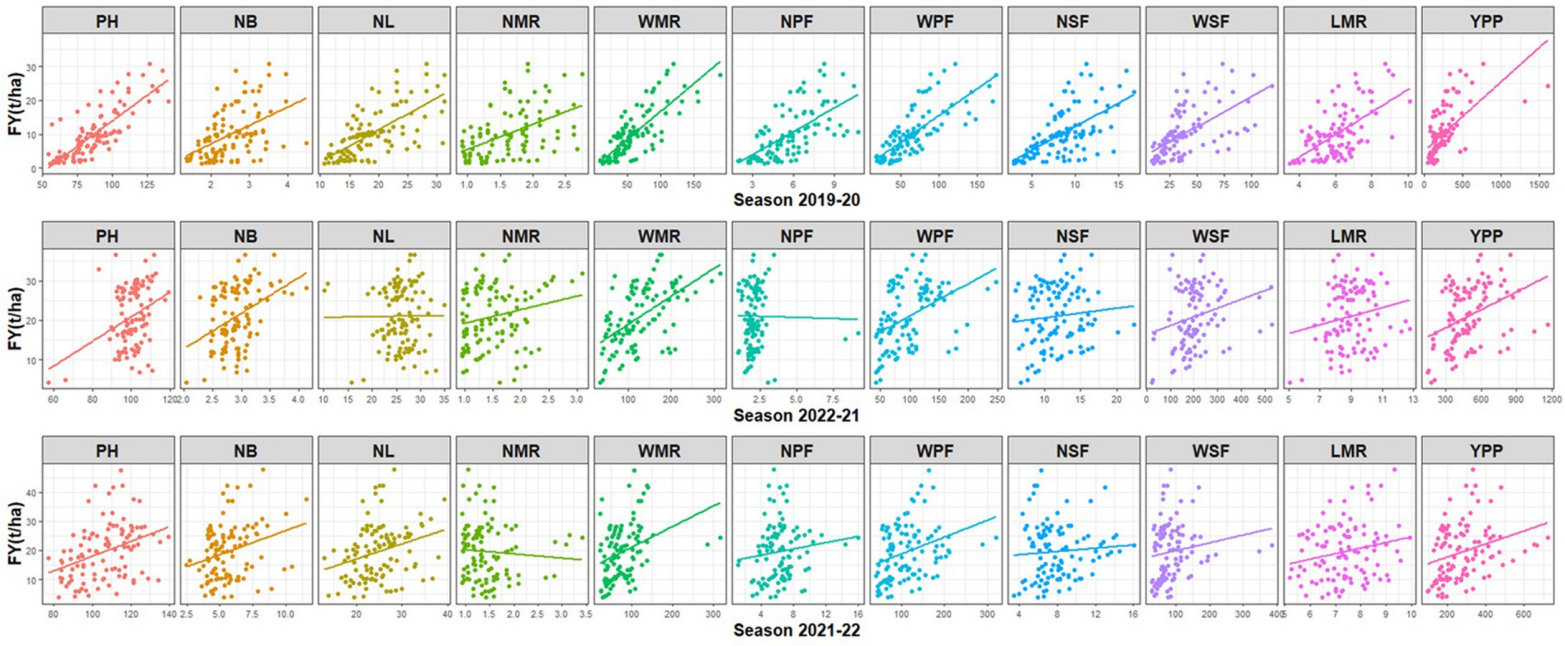
Graph displaying contributions of different traits to the fresh yield variation. [Here the abbreviation represents as: PH = Plant Height; NB = Number of branches; NL = Number of leaves; NMR = Number of mother rhizome; WMR = Weight of mother rhizome; NPF = Number of primary fingers; WPF = Weight of primary finger; NSF = Number of secondary fingers; WSF = Weight of secondary finger; MRL = Length of mother rhizome; YPP = Yield per plant; FY = Fresh yield].

The traits association shows the relationship among the traits, while regression of the independent traits towards the dependent traits revealed the extent of the relation as well as the contribution to the dependent trait’s variation i.e., fresh yield in the case of the present study. When all season data was considered, the multiple linear regression analysis revealed that PH, NMR, and WMR were the most contributory traits. The studied traits were responsible for 37–79% of the FY variability (Table 3). The regression of individual traits on FY is depicted in Fig 1. The graph shows the influence of studied traits on the grain yield variation in different seasons. During the year 2019–20, all the traits exhibited positive contributions to the FY variation. Furthermore, NL and NPF contributed little or nothing in the year 2020–21. While in the year 2021–22, NMR and NSF did not account for fresh yield.

**Table 3:** Contribution of different traits on FY variation obtained from multiple linear regression.

Traits		PH	NB	NL	NMR	WMR	NPF	WPF	NSF	WSF	LMR	YPP	MRL	
													r^2^	P-value
2019–20	b	0.12	-0.98	0.21	1.05	0.01	0.07	0.06	0.26	-0.02	0.66	0.00	79	<0.000
	P-value	0.00	0.36	0.21	0.30	0.60	0.78	0.00	0.09	0.35	0.10	0.19		
2020–21	b	0.06	8.12	-0.05	-4.46	0.08	0.19	0.00	-0.92	0.01	-1.27	0.01	52	<0.000
	P-value	0.60	0.00	0.77	0.04	0.00	0.77	0.87	0.00	0.34	0.04	0.03		
2021–22	b	0.18	0.90	0.01	-6.22	0.16	-0.50	0.06	-0.31	0.06	-1.38	-0.04	37	<0.000
	P-value	0.01	0.24	0.97	0.01	0.01	0.42	0.10	0.63	0.23	0.20	0.27		

PH = Plant Height; NB = Number of branches; NL = Number of leaves; NMR = Number of mother rhizome; WMR = Weight of mother rhizome; NPF = Number of primary fingers; WPF = Weight of primary finger; NSF = Number of secondary fingers; WSF = Weight of secondary finger; MRL = Length of mother rhizome; YPP = Yield per plant. MRL = Multiple linear regression; b = Slope; r^2^ = Coefficient of determination.

### 3.5 Clustering pattern

**Fig 2:**
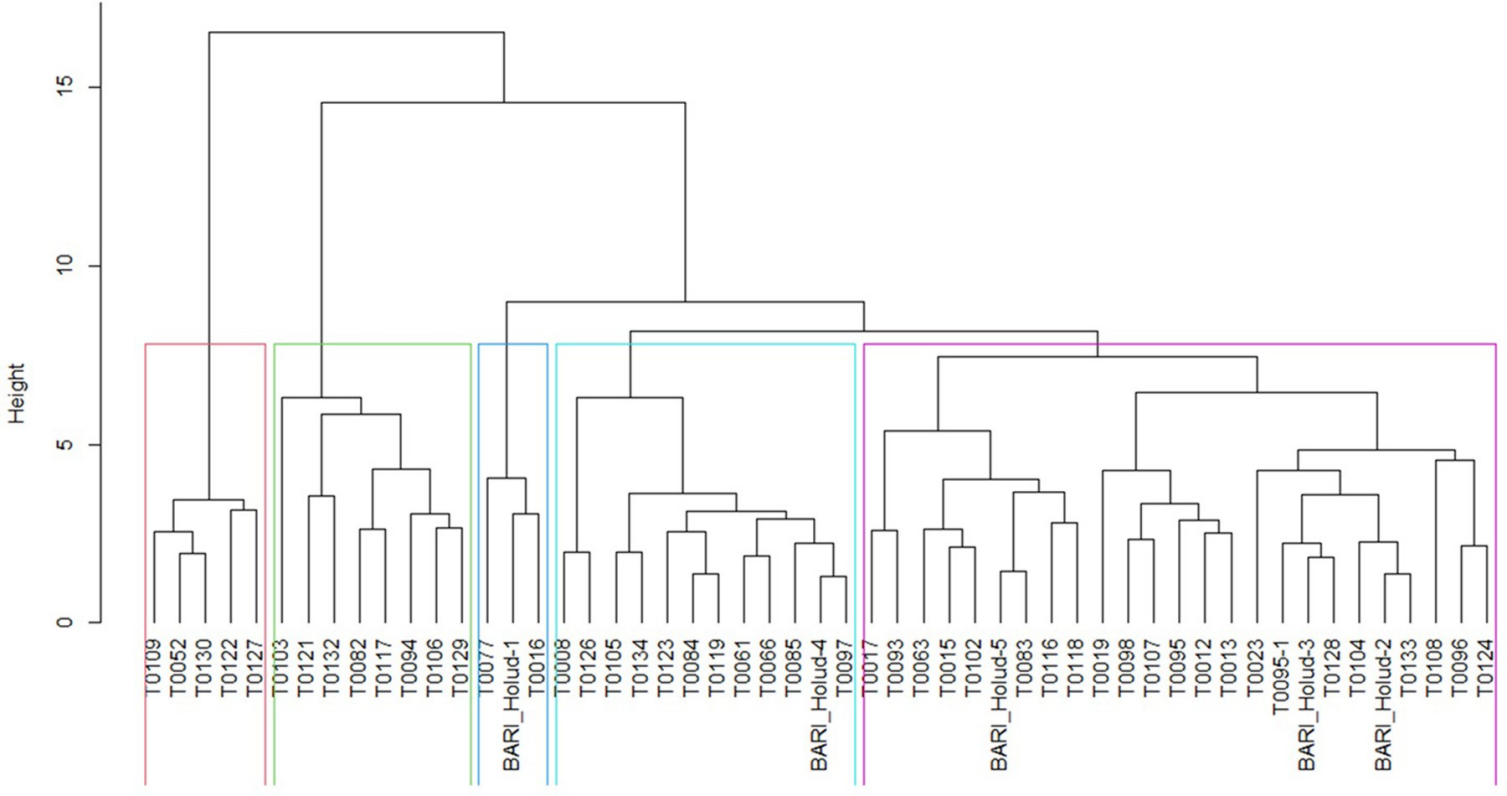
Dendrogram showing the grouping of turmeric genotypes based on all the trait’s performances over the years.

Clustering of genotypes based on a similarity index is a useful technique commonly employed in quantitative genetics. In this study, all the genotypes were categorized into five distinct groups where cluster II possessed the maximum number of genotypes (25, 47.17%) followed by cluster III (12), V (8) and IV (5) while cluster I had the least number (3, 5.66%) (Table 4 and Fig 2). The schematic dendrogram also shows the divergence pattern of the studied genotypes (Fig 2). Inter-cluster distances express the existing diversity among the genotypes between the clusters whereas intra-cluster distances represent the diversity among genotypes within the clusters. The maximum intra-cluster distance was observed among the genotypes of group V (6.75) and the minimum was for group I (3.82) (Table 5). The maximum dissimilarity between cluster genotypes was found for clusters IV and V (11.83); while the minimum was found for genotypes between clusters II and III (6.31).

**Table 4:** Grouping of turmeric genotypes based on different traits performances over the years.

Cluster	Genotypes (no.)	Percentage (%)	Genotypes
I	3	5.66	BARI_Holud-1, T0016 and T0077
II	25	47.17	BARI_Holud-2, BARI_Holud-3, BARI_Holud-5, T0012, T0013, T0015, T0017, T0019, T0023, T0063, T0083, T0093, T0095, T0095-1, T0096, T0098, T0102, T0104, T0107, T0108, T0116, T0118, T0124, T0128 and T0133
III	12	22.64	BARI_Holud-4, T0008, T0061, T0066, T0084, T0085, T0097, T0105, T0119, T0123, T0126 and T0134
IV	5	9.43	T0052, T0109, T0122, T0127 and T0130
V	8	15.09	T0082, T0094, T0103, T0106, T0117, T0121, T0129 and T0132

**Table 5:** Intra (diagonal) and inter-cluster distances of different cluster.

Cluster	I	II	III	IV	V
I	3.82				
II	7.62	5.77			
III	6.57	6.31	4.71		
IV	7.61	7.99	8.83	3.20	
V	8.98	8.82	7.20	11.83	6.75

The mean values of clusters for yield and its various contributing traits are presented in Table 6. Regarding plant height, dwarf genotypes were primarily grouped in cluster VI (83.61), followed by cluster I (87.97). In contrast, the tallest genotypes were observed in cluster V (110.15 cm). The most number of fingers (primary-5.6 and secondary 10.69) were produced by the genotypes of Cluster V. Cluster V also emerged as the top performer in terms of fresh yield, producing 24.50 t/ha, followed by cluster III at 18.89 t/ha. Conversely, the lowest yield was recorded in genotypes of cluster IV (6.91 t/ha).

**Table 6:** Mean values of different clusters for different studied traits.

Cluster	PH	NB	NL	NMR	WMR	NPF	WPF	NSF	WSF	LMR	YPP	FY
I	87.97	3.31	20.85	1.92	56.98	4.36	96.19	9.00	184.25	6.18	333.84	14.46
II	96.54	3.76	22.96	1.38	78.86	4.73	84.42	9.23	95.43	7.58	296.28	15.69
III	100.46	3.54	22.67	1.56	105.75	4.91	104.26	10.06	128.58	7.76	390.32	18.89
IV	83.61	3.04	19.54	1.33	39.34	3.05	45.86	6.30	65.52	6.25	182.31	6.91
V	110.15	4.20	26.95	1.88	131.49	5.60	122.51	10.69	131.34	7.85	484.08	24.50

PH = Plant Height; NB = Number of branches; NL = Number of leaves; NMR = Number of mother rhizome; WMR = Weight of mother rhizome; NPF = Number of primary fingers; WPF = Weight of primary finger; NSF = Number of secondary fingers; WSF = Weight of secondary finger; MRL = Length of mother rhizome; YPP = Yield per plant; FY = Fresh yield

Considering all analyzed characteristics, cluster V exhibited exemplary performance, characterized by a relatively taller plant, numerous branches, leaves, primary finger, secondary finger, heaviest mother rhizome and primary finger, and maximum yielding ability. Genotypes of Cluster I demonstrated a notable performance for different yield contributing traits and a reasonable yield capacity. While genotypes of cluster I exhibited the maximum number of mother rhizomes and heaviest secondary fingers. Most of the selected genotypes fall into cluster V.

### 3.6 Geometric Adaptability Index (GAI)

The GAI is used to measure how well a genotype can adapt and how much better it is than other genotypes (Table 7). The genotypes with the lowest values have been ranked in ascending order. Based on plant height, variety T0117 (116.34) had the best geometric adaptability index (S12 Table). T0129 (116.10), T0103 (112.76), and T0121 were better adaptable varieties (110.47). T0127 was the variety with the lowest geometric adaptability (70.33). The varieties with the lowest geometric adaptability were T0077 (79.41), T0108 (79.91), and T0122 (80.02).

**Table 7:** Ranking and mean ranking of studied genotypes based on the geometric adaptability index.

GEN	PH	NB	NL	NMR	WMR	NPF	WPF	NSF	WSF	LMR	YPP	FY	MR	R.MR	Occurrence* (In top 10)
BARI Holud-1	40	48	41	5	47	29	20	33	3	45	24	21	29.67	34	2
BARI Holud-2	43	47	31	32	39	36	42	28	26	25	47	39	36.25	44	-
BARI Holud-3	28	35	34	9.5	21	43	27	29	28	43	33	24	29.54	33	1
BARI Holud-4	11	23.5	25	22	13	20	11	12	13	7	20	14	15.96	10	1
BARI Holud-5	16	21	14	26	42	40	35	34	43	47	38	25	31.75	38	-
T0008	24	43	38	43.5	12	18	13	13	15	19	21	35	24.54	22	-
T0012	41	46	43	15	34	13	12	6	27	41	27	37	28.50	30	1
T0013	48	29	35	36	45	25	30	10	32	23	40	48	33.42	40	1
T0015	17	6	20	43.5	19	33	28	30	33	11	29	8	23.13	18	2
T0016	39	44	24	8	40	31	34	7	2	48	15	29	26.75	26	3
T0017	20	33	8	53	37	28	37	11	17	4	42	31	26.75	27	2
T0019	47	3	16	25	22	1	22	8	42	44	19	11	21.67	16	3
T0023	30	53	23	47	33	51	41	15	8	8	9	30	29.00	36	3
T0052	49	51	44	18	46	52	49	53	52	51	50	51	47.17	51	-
T0061	13	15	29	22	5	17	8	41	38	17	14	9	19.00	15	3
T0063	23	7	22	49	25	30	31	40	41	16	36	32	29.33	32	-
T0066	27	23.5	32	41	17	24	15	27	20	46	11	13	24.71	23	1
T0077	52	40	51	3	48	37	25	50	25	53	31	49	38.67	45	1
T0082	7	2	10	9.5	2	32	7	1	11	2	3	5	7.63	3	10
T0083	32	10	9	30	38	41	48	35	44	39	43	40	34.08	42	2
T0084	14	17	19	6	31	7	16	22	19	21	28	12	17.67	11	2
T0085	18	13	11	19	8	9	10	17	16	14	5	10	12.50	7	5
T0093	35	9	2	52	32	5	18	26	40	1	12	27	21.58	15	4
T0094	6	12	5	2	14	3	29	32	24	31	22	3	15.25	9	5
T0095	36	30	18	22	50	12	47	20	31	42	44	42	32.83	39	-
T0095-1	31	25	42	40	35	46	39	48	45	40	32	43	38.83	46	-
T0096	12	27	53	48	16	38	40	46	30	6	39	16	30.92	37	1
T0097	8	34	26	14	11	16	23	18	21	26	16	22	19.58	14	1
T0098	21	28	30	33	29	6	21	31	35	35	30	34	27.75	29	1
T0102	19	4	39	31	28	19	32	25	23	29	34	15	24.83	24	1
T0103	3	1	1	27	23	11	1	37	22	10	8	2	12.17	6	7
T0104	45	41	36	42	43	48	45	44	50	13	41	50	41.50	48	-
T0105	33	38	28	16	20	26	33	23	9	18	17	33	24.50	21	1
T0106	5	5	3	7	6	14	19	16	5	27	2	6	9.58	4	8
T0107	26	19	12	36	26	2	24	4	48	20	49	26	24.33	20	2
T0108	51	42	50	38	41	8	4	47	18	22	18	19	29.83	36	2
T0109	42	49	48	50	51	53	53	52	51	50	53	53	50.42	53	-
T0116	22	8	6	29	27	42	44	38	34	33	23	20	27.17	28	2
T0117	1	11	17	4	4	10	6	14	10	3	10	1	7.58	2	9
T0118	10	18	15	46	36	47	14	36	14	38	26	17	26.42	25	1
T0119	9	20	27	11.5	18	35	26	24	36	15	35	18	22.88	17	1
T0121	4	26	7	1	1	4	2	5	1	30	1	7	7.42	1	10
T0122	50	31	37	51	53	49	50	45	46	52	37	46	45.58	49	-
T0123	15	50	40	20	7	23	46	39	39	24	25	28	29.67	35	1
T0124	29	36	47	45	24	21	38	42	37	5	46	44	34.50	43	1
T0126	44	37	33	34	9	44	5	2	4	28	13	36	24.08	19	4
T0127	53	45	52	36	52	39	52	51	53	49	52	52	48.83	52	-
T0128	34	32	46	28	30	27	36	19	29	34	45	45	33.75	41	-
T0129	2	22	13	17	15	22	9	21	7	36	7	4	14.58	8	5
T0130	46	52	49	24	49	50	51	49	49	37	51	47	46.17	50	-
T0132	25	16	4	13	3	15	3	3	6	12	4	23	10.58	5	6
T0133	37	39	45	39	44	34	43	43	47	32	48	38	40.75	47	-
T0134	38	14	21	11.5	10	45	17	9	12	9	6	41	19.46	13	4

PH = Plant Height; NB = Number of branches; NL = Number of leaves; NMR = Number of mother rhizome; WMR = Weight of mother rhizome; NPF = Number of primary fingers; WPF = Weight of primary finger; NSF = Number of secondary fingers; WSF = Weight of secondary finger; MRL = Length of mother rhizome; YPP = Yield per plant; FY = Fresh yield; MR = Mean rank; R.MR = Rank of MR

* Total number of times a genotype is present within the top ten at different ranking

The genotype T0121 (142.05) had the highest geometric adaptability based on mother rhizome weight, followed by T0082 (129.13), T0132 (123.29), and T0117 (114.24). Contrary to that T0122 (25.51) had the lowest geometric adaptability followed by T0127 (25.69), T0109 (34.16), and T0095 (40.74). Based on primary finger weight, T0103 (162.56) had the highest geometric adaptability, followed by T0121 (139.99) and T0132 (123.07); T0109 (36.56) had the lowest geometric adaptability, followed by T0127 (39.16) and T0130 (39.96). On the other hand, in terms of secondary fingers weight, the most geometrically adaptable variety was T0121 (140.93g, followed by T0016 (134.52) and BARI Holud-1 (127.19); the least adaptable was T0127 (29.12), followed by T0052 (40.18), T0109 (41.87), and T0104 (43.33).

Based on the yield per plant, the variety with the most geometric adaptability was T0121 (601.46), followed by T0106 (460.63) and T0082 (443.08). The variety with the lowest geometric adaptability, according to yield per plant, was T0127 (134.35), followed by T0130 (156.90), and T0052 (157.60). Likewise, according to fresh yield, the variety with the highest geometric adaptability was T0117 (26.44), followed by T0129 (25.47), and T0094 (25.79). The variety with the lowest geometric adaptability, according to fresh yield, was T0109 (3.78), and the following three varieties were T0127 (3.98), T0052 (5.56), and T0104 (6.34).

Genotypes were ranked based on GAI indices of different traits to identify better ones (Table 7). Based on individual traits, genotypes were ranked, where differences in ranks for different traits were obtained. Therefore, a mean ranking (MR) was estimated for all the GAI indices. Many genotypes were found in the higher order in the MR of various traits, with varied outcomes also present. A re-ranking of MR was calculated to choose superior genotypes with good yield and better trait performances. Genotypes’ position within the top ten was also counted. The genotypes that appeared a minimum of 5 times in the top ten were regarded as superior-performing genotypes. According to the FY ranking, the genotype T0117 came in first place, followed by T0103 and T0094. The genotypes T0121, T0117, T0082, and T0106 were found to have the best performance based on the mean rank. Based on yielding capacity and overall ranking, the genotypes T0015, T0061, T0082, T0085, T0094, T0103, T0106, T0117, T0121, T0129 and T0132 were determined to be the superior group.

### 3.7 Genotype x year interaction for fresh yield

Genotype × Year interaction analysis was carried out depending on fresh yield observations at different years. Substantial variation (p≤0.01) was recorded for turmeric genotypes in the combined analysis of variance (Table 8) which indicates the difference in the genotype’s response at different years. The highest portion (40.80%) of the total sum of square was explained by the genotypic effect which indicated the presence of ample genetic variability among the studied genotypes and the possibility of selection for stable high-yielding genotypes. The year was the least sources of variation and contributed a small portion (26.07%) to the total sum of squares. But the significant difference was spotted for genotypes × years interaction suggesting that rhizome yield of genotypes varied across the years and also reflects the existence of year effects in the genotype x year interaction. A high percentage (32.05%) to the total sum of squares for genotype x year interaction displays the significance of this source of variation and also implicates truncated effectiveness of indirect selection for potential genotype disregarding the genotypes × Years interaction. Genotype year interaction has a role in the stability of the tested genotypes.

**Table 8:** Genotype-Year interaction ANOVA for fresh yield studied during three years.

Source of variation	Degrees of Freedom	Sum Squares	Mean Squares	% Total SS
Rep	1	3.4	3.4	0.01
Genotype	52	11675.69	224.53^**^	40.80
Year	2	7458.75	3729.38^**^	26.07
Genotype x year	104	9171.76	88.19^**^	32.05
Residuals	156	293.47	1.88	

** 1% level of significance; SS = Sum of squares

## 4. Discussion

Fifty-three turmeric cultivars were evaluated in the current study to determine heritability and variance based on twelve morpho-physiological parameters. For all of the traits, the variations due to genotypes under this study were enormously significant (p<0.01). This could be a result of genetic inheritance in the varieties. Many different methods have been employed which can help to find an underlying cause and describe levels of genetic variation [29]. This variation designates that depending on these traits’ selection can be done to detect the promising genotypes. Significant differences among turmeric cultivars were found in yield-contributing parameters such as the number of fingers/rhizome, plant height, curcumin content, the weight of the rhizome per plant and rhizome yield in an earlier study [30]. A study conducted by Mishra et al. [31] and Lal et al. [32] found significant differences in the morphological traits of 135 turmeric accessions collected from different parts of India. Similarly, a separate study conducted by Akter et al. [33] and Tanvir et al. [34] found significant variation in the biochemical traits of turmeric cultivars grown in Bangladesh, including curcumin content and antioxidant activity.

The genotypic coefficient of variation (GCV) and phenotypic coefficient of variation (PCV) are important parameters used to estimate the extent of variability and the potential for genetic improvement. The high genotypic coefficient of variation values of traits was also reflected in a wide range of mean values. In general, estimates of the genotypic coefficient of variation are lower than those of the phenotypic coefficient of variation found in this study, indicating an environmental influence. However, the differences between the phenotypic and genotypic coefficients of variation for all corresponding traits were small, indicating that these traits are less susceptible to environmental influences and thus more heritable in nature. Estimates of the genotypic coefficient of variation provide good implications for genetic potential in crop improvement through selection [35]. Different studies conducted by Vinodhini et al. [36] and Dev and Sharma [37] and reported high genotypic coefficient of variation and phenotypic coefficient of variation for rhizome yield, curcumin content, and essential oil content. Similarly, investigations conducted by Khan et al. [38], Terfa and Gurmu [39] reported high genotypic coefficient of variation and phenotypic coefficient of variation for rhizome yield, curcumin content, and essential oil content. These findings of high genotypic coefficient of variation and phenotypic coefficient of variation indicate that these traits have a high potential for genetic improvement. A study executed by Vimal et al. [40] reported a high genotypic coefficient of variation for yield and its components, such as rhizome weight and number of fingers, in turmeric. Therefore, there is an opportunity for selection based on these traits [41].

High heritability accompanied by high genetic advance was estimated for plant height, weight of mother rhizome, weight of primary finger, weight of secondary finger, yield per plant and fresh yield in different years. High heritability and genetic advance for most of the traits indicate that genetic factors play a significant role in determining this trait suggesting the potential for genetic improvement through selection. A study performed by Kundu et al. [14] reported high heritability and genetic advancement for rhizome yield, curcumin content, and essential oil content. Similar findings were also observed in other research [37, 42–50]. In contrast, the number of branches, number of leaves, number of mother rhizomes, number of primary fingers, number of secondary fingers, and length of mother rhizome etc. showed high heritability along with moderate to low genetic advance in all of these seasons. Similar findings of high heritability with moderate to low genetic advance were also reported in different studies on turmeric [37, 41, 51] and on rice [52]. These findings indicated that these traits are under genetic control and can be improved through selection. Neetu et al. [50] also reported moderate to high heritability and genetic advancement for most of the traits, suggesting the potential for genetic improvement through selection.

In this investigation, we found that the variety with the most secondary fingers did not have the heaviest secondary fingers, and the opposite was true for the variety with the fewest secondary fingers. The lowest number of primary finger genotypes produced the lowest yield. The mother rhizome-producing genotype’s mother rhizome weight was the highest and also the same case happened for the lowest number of mother rhizome producing variety with its weight. It indicated that cultivars had a proportional influence over the weight of rhizomes to the total yield [53].

The geometric adaptability index (GAI) is a way to measure how well a cultivar can grow in different kinds of environments. It is based on the concept of geometric mean yield (GMY), which considers both the mean yield and the variability of the yield across different environments. A cultivar with a high geometric adaptability index is considered to be more adaptable to different environments than a cultivar with a low geometric adaptability index. According to the ranking of genotypes by GAI, genotypes with a high geometric adaptability index (low ranks) are preferred [24, 54]. In this study, differential GAI ranks were found for different genotypes for different traits. Based on mean rank (MR) considering all traits performances, a few genotypes were identified as better performers. Genotype ranking is a useful tool for selecting the best turmeric genotypes that have high yield potential and are adapted to the prevailing environmental conditions [4]. These findings indicate that the selection of stable genotypes is important for the genetic improvement of turmeric. In a study, it was concluded that the cultivars had varying levels of adaptability, and the cultivars with the highest geometric adaptability index values were found to be more stable across different locations and had higher mean yields compared to the cultivars with lower geometric adaptability index values in maize [55] and in safflower [56].

Correlation and regression analyses are important tools to find out how different traits are linked. Correlation coefficients between different traits have been calculated at the phenotypic and genotypic levels. In general, genotypic correlation coefficients are higher than phenotypic correlation coefficients, this suggests that associations were not strongly influenced by environmental factors [33] and also indicates strong inherent associations between traits [37]. A study reported a significant positive correlation between rhizome yield and plant height, and between rhizome yield and curcumin content [57]. Similarly, a study conducted by [58] reported a positive correlation between rhizome yield and curcumin content. A positive correlation between rhizome yield and plant height on turmeric genotypes was found in earlier studies [59, 60]. Singh et al. [61] also found positive and significant correlations between yield and its components, indicating the importance of these traits in yield improvement. These findings indicate that the selection for these traits could result in simultaneous improvement.

Regression analysis can provide insights into the relationships between different turmeric traits, which can help in selecting and improving desirable traits. Multiple linear regression is used to find linear relationships between one or more predictor variables and a dependent variable. In linear regression, fresh yield was determined by the variables number of leaves, length of mother rhizome, numbers of primary finger, plant height, weight of mother rhizome, weight of primary finger and yield per plant. Netsere and Weldemichael [62] found that plant height, number of leaves, and rhizome length were significant predictors of rhizome yield, suggesting that these traits can be used for selecting high-yielding genotypes. Multiple linear regression can effectively be used to find the most contributory traits to the fresh yield variation in turmeric [63, 64]. It was observed that with a 1 q/ha increase in dry matter, there would be a 9.031 q/ha increase in fresh yield [65]. In a different study, it was reported that rhizome weight had a major impact on final fresh yield variation [48].

Diversity analysis is used to determine the degree of genetic diversity within a population or group of individuals. In the present study, higher levels of diversity were observed for the studied genotypes and they fall into five different clusters based on the traits studied. A study led by Kumari et al. [66] used clustering analysis to group turmeric accessions collected from different parts of India based on their morphological and biochemical characteristics. In earlier research implemented by Ashraf et al. [67] and Dudekula et al. [58] found high levels of genetic diversity among turmeric accessions collected from different parts of India. Khan et al. [68] reported a high degree of diversity among turmeric accessions based on morphological and molecular markers. Similarly, Bahadur et al., [69] reported that the turmeric accessions were grouped into different clusters based on morphological and molecular markers. A study executed by Verma et al. [70] evaluated the genetic diversity of turmeric genotypes using molecular markers.

Genetic diversity in turmeric was also studies in earlier studies and found the studied genotypes fall into different clusters based on different studied traits [70–72]. Genotypes collected from the same source didn’t always fall into the same group, again differentially derived ones were included in the same group. It’s indicated that the geographical distribution of the genotypes doesn’t confirm the homogeneity [58, 73]. Divergent groups were formed based on genotype performances in earlier studies [58, 74]. Among the five clusters, cluster II possessed the maximum number of accessions indicating that about half of the studied genotypes are similar in performance. In previous research, morphologically similar genotypes were found to be included in the same cluster [58, 70]. In the present study, higher cluster means for different traits were observed in cluster V, similar to this higher means for different traits were also reported in a single cluster in earlier studies [58, 72]. The findings of this study can be applied to the development of turmeric breeding programs. To fulfil the rising demand for turmeric for industrial and pharmaceutical uses, it will be necessary to boost the production of curcumin, oleoresin, and essential oils through breeding programs in the future [71]. Genotype environment interaction (GEI) is an important parameter used to evaluate the stability and adaptability of genotypes to different environments. A study conducted by [4] reported a significant GEI for rhizome yield and curcumin content. Tavares et al. [75] reported significant GEI for yield and its components in turmeric, indicating the need for location-specific selection.

## 5. Conclusion

The present study on turmeric genotypes evaluated over three consecutive seasons identified ten superior performers based on both fresh yield and geometric adaptability indices. Genotype T0015 stood out as the top yielder at 28.04 t/ha, surpassing both the existing cultivar and nine other genotypes. Nine genotypes consistently outperformed the current best cultivar in yield. Sufficient variability was present in the tested genotypes for the studied traits suggesting potential for selection in future turmeric breeding programs. Positive and significant correlations between fresh yield and key traits, such as plant height and weight of mother rhizome, underscored the importance of these traits. Multiple linear regression highlighted plant height, numbers of mother rhizome, and weight of mother rhizome as major contributors, explaining 37–79% of the variability in fresh yield. Cluster analysis grouped genotypes into five clusters, revealing distinct relationships. The geometric adaptability index (GAI) ranked T0117 as the top-performing genotype, followed by T0103 and T0094. T0121, based on mean ranks, emerged as the best performer, followed by T0117, T0082, and T0106. The top ten genotypes, including T0015, T0061, T0082, T0085, T0094, T0103, T0106, T0117, T0121, and T0129, are recommended for further evaluation across diverse locations, highlighting their potential to enhance turmeric yield and contribute to the improvement of this globally cultivated spice crop. The selected genotypes, when grown for mass production, could address existing demand. Future studies on qualitative aspects, such as curcumin content, would offer additional insights for genotype selection. Therefore, the study provides valuable knowledge on turmeric productivity, motivating producers and potentially improving overall farmer livelihoods.

## Supporting information

S1 Table List of genotypes with local name and source used in the present study. (DOCX)

S2 Table Prevailed weather condition during the study at the experimental site during 2019–2020, 2020–2021 and 2021–2022. (DOCX)

S3 Table Physical properties of initial soil of the experimental plot, 2019–2020, 2020–2021 and 2021–2022. (DOCX)

S4 Table Chemical properties of initial and post-harvest soil of the experimental site, 2019–2020, 2020–2021 and 2021–2022. (DOCX)

S5 Table Mean performance of 53 genotypes of turmeric grown during the year of 2019–22. (DOCX)

S6 Table Mean performance of 53 genotypes of turmeric grown during the year of 2019–20. (DOCX)

S7 Table Mean performance of 53 genotypes of turmeric grown during the year of 2020–21. (DOCX)

S8 Table Mean performance of 53 genotypes of turmeric grown during the year of 2021–22. (DOCX)

S9 Table Association of studied traits based on the performances during the year of 2019–20. (DOCX)

S10 Table Association of studied traits based on the performances during the year of 2020–21. (DOCX)

S11 Table Association of studied traits based on the performances during the year of 2021–22. (DOCX)

S12 Table Geometric adaptability index of the studied genotypes evaluated over three consecutive years. (DOCX)

## References

[1] Sasikumar B. Genetic Resources of Curcuma: Diversity, Characterization and Utilization. Plant Genet. Resour. 2005, 3, 1479–2621, doi: 10.1079/PGR200574

[2] Chattopadhyay I.; Biswas K.; Bandyopadhyay U.; Banerjee R. Turmeric and Curcumin: Biological Actions and Medicinal Applications. Curr Sci 2004, 87, 44–53.

[3] Singh B.; Pathak K.; Ramakrishna Y. Underutilized Vegetable Crops and Spices of Mizoram: Needs Exploration and Utilization. In; 2013; pp. 217–232.

[4] Anandaraj M.; Prasath D.; Kandiannan K.; Zachariah T.J.; Srinivasan V.; Jha A.K.; et al. Genotype by Environment Interaction Effects on Yield and Curcumin in Turmeric (Curcuma longa L.). Ind. Crops Prod. 2014, 53, 358–364, doi: 10.1016/j.indcrop.2014.01.005

[5] Mishra A.; Kumar R.; Tyagi A.; Kohaar I.; Hedau S.; Bharti A.C.; et al. Curcumin Modulates Cellular AP-1, NF-KB, and HPV16 E6 Proteins in Oral Cancer. Ecancermedicalscience 2015, 9, 525, doi: 10.3332/ecancer.2015.525 25932049 PMC4407748

[6] Joshi P.; Joshi S.; Semwal D.K.; Bisht A.; Sharma S.; Dwivedi J. Chemical Composition, Antioxidative and Antimicrobial Activities of Turmeric Spent Oleoresin. Ind. Crops Prod. 2021, 162, 113278, 10.1016/j.indcrop.2021.113278

[7] Sharifi-Rad J., Rayess Y.E., Rizk A.A., Sadaka C., Zgheib R., Zam W., et al. Turmeric and its major compound curcumin on health: bioactive effects and safety profiles for food, pharmaceutical, biotechnological and medicinal applications. Front. Pharmacol. 2020, 11, 1021, doi: 10.3389/fphar.2020.01021 33041781 PMC7522354

[8] Ravindran P.N.; Babu K.N.; & Sivaraman K., Turmeric: The Genus Curcuma;Ed.; 1st ed.; CRC Press, 2007.

[9] Agricultural Market Intelligent Centre (PJTSAU) Turmeric Outlook; 2022.

[10] Chandra R.; Desai A.R.; Govind S.; Gupta P.N. Metroglyph Analysis in Turmeric (Curcuma longa L.) Germplasm in India. Sci. Hortic. (Amsterdam). 1997, 70, 211–222, doi: 10.1016/S0304-4238(97)00036-8

[11] Gupta A.K.; Mishra R.; Lal R.K. Genetic Resources, Diversity, Characterization and Utilization of Agronomical Traits in Turmeric (Curcuma Longa L.). Ind. Crops Prod. 2015, 77, 708–712, 10.1016/j.indcrop.2015.09.030

[12] BBS Yearbook of Agricultural Statistics-2021. Yearb. Agric. Stat. 2022, 33, 228.

[13] BBS Household Income and Expenditure Survey; Bangladesh Bureau of Statistics (BBS), Statistics and Informatics Division (SID), Ministry of Planning, Bangladesh, 2019;

[14] Kundu P., Ghosh S., Ghosal A., Saha M. and Mukherjee A., Comparative Assessment of Popular Turmeric Cultivars in Coastal Zone of South 24 Parganas District of West Bengal under STI Technique. J. 2019, 8, 2601–2605.

[15] Holttum R.E. The Zingiberaceae of the Malay Peninsula. Gard. Bull. Singapore 1950, 13, 1–249.

[16] R Core Team R: A Language and Environment for Statistical Computing. 2021.

[17] Mendiburu, F. De Package ‘ Agricolae.’ R Packag. version 1.2–3 2015, doi:10.2307/2411227>.

[18] Lush J.L. Heritability of Quantitative Characters in Farm Animals. Hereditas 1949, 35.S1, 356–375, doi: 10.1111/j.1601-5223.1949.tb03347.x

[19] Robinson H.F.; Comstok R.E.; Harvey P.H. Estimates of Heritability and the Degree of Dominance in Corn. Agron. J. 1949, 41, 353–359.

[20] Alvarado, G.; López, M.; Vargas, M.; Pacheco, A.; Rodr\’\iguez, F.; Burgueño, J.; et al. META-R (Multi Environment Trial Analysis with R for Windows) Version 6.01. Hdl: 11529/10201; International Maize and Wheat Improvement Center, 2016; Vol. 20;.

[21] Bates, D.; Maechler, M.; Bolker, B.; Walker, S. Lme4: Linear Mixed-Effects Models Using S4 Classes. R Package Version 1.1–8. R 2015.

[22] Mahalanobis P.C. On the Generalized Distance in Statistics. Proc. Natl. Inst. Sci. 1936, 2, 49–55.

[23] Rao C.R. Advanced Statistical Methods in Biometric Research.; John Wiley & Sons, Inc., New York,: Oxford, England, 1952;

[24] Mohammadi R.; Amri A. Comparison of Parametric and Non-Parametric Methods for Selecting Stable and Adapted Durum Wheat Genotypes in Variable Environments. Euphytica 2008, 159, 419–432, doi: 10.1007/s10681-007-9600-6

[25] Mengistu B.; Abu M. Evaluation of Stability Parameters for the Selection of Stable and Superior Sunflower Genotypes. Cogent Food Agric. 2023, 9, 2275406, doi: 10.1080/23311932.2023.2275406

[26] Aghogho C.I.; Eleblu S.J.Y.; Bakare M.A.; Kayondo I.S.; Asante I.; Parkes E.Y.; et al. Genetic Variability and Genotype by Environment Interaction of Two Major Cassava Processed Products in Multi-Environments. Front. Plant Sci. 2022, 13, 974795, doi: 10.3389/fpls.2022.974795 36325542 PMC9618686

[27] VERMA A. Wheat Genotypes Evaluated for GxE Interactions in Central Zone of the Country by AMMI Analysis. Ann. Plant Soil Res. 2021, 23, 341–345, doi: 10.47815/apsr.2021.10081

[28] Olivoto T.; Lúcio A.D.C. Metan: An R Package for Multi-Environment Trial Analysis. Methods Ecol. Evol. 2020, 11, 783–789, doi: 10.1111/2041-210X.13384

[29] Fetzner Jr, J.; Crandall, K. Genetic Variation. In Nature; 2001; Vol. 238, pp. 291–326 ISBN 0-632-05431-X.

[30] Shashidhar T.R.; Sulikeri G.S. Effect of Spacing and Nitrogen Levels on Nutrient Uptake and Yield of Turmeric. Karnataka J. Agric. Sci. 1996, 9, 649–656.

[31] Mishra R.; Gupta A.K.; Lal R.K.; Jhang T.; Banerjee N. Genetic Variability, Analysis of Genetic Parameters, Character Associations and Contribution for Agronomical Traits in Turmeric (Curcuma Longa L.). Ind. Crops Prod. 2015, 76, 204–208, 10.1016/j.indcrop.2015.06.049

[32] Lal M.; Munda S.; Begum T.; Gupta T.; Paw M.; Chanda S.K.; et al. Identification and Registration for High-Yielding Strain through ST and MLT of Curcuma Caesia Roxb. (Jor Lab KH-2): A High-Value Medicinal Plant. Genes (Basel). 2022, 13. doi: 10.3390/genes13101807 36292691 PMC9601691

[33] Akter, A.; Hasan, M.J.; A., L.; Kulsum, U.; Biswas, P.; Rahman, M.; et al. Genetic Variability, Heritability, Correlation and Path Coefficient Studies for Yield and Yield Components of Some Promising Rice Hybrids. Bangladesh Rice J. 2020, 23, 27–34, doi:10.3329/brj.v23i2.48245.

[34] Tanvir E.M.; Hossen M.S.; Hossain M.; Afroz R.; Gan S.; Khalil M.; et al. Antioxidant Properties of Popular Turmeric (Curcuma longa L.) Varieties from Bangladesh. J. Food Qual. 2017, 2017, 1–8, doi: 10.1155/2017/8471785

[35] Johnson H.W.; Robinson H.F.; Comstock R.E. Estimates of Genetic and Environmental Variability in Soybeans. Agron. J. 1955, 270, 314–318, doi: 10.2134/agronj1955.00021962004700070009x

[36] Vinodhini, V.; Balaraman, S.; S, B.; Suresh, R. Evaluation of Turmeric (Curcuma LongaL.) Genotypes for Yield and Curcumin Content. J. Agric. Ecol. 2019, 07, 88–95, doi:10.53911/JAE.2019.7109.

[37] Dev H.; Sharma V. Genetic Variability in Turmeric (Curcuma longa L.). Int. J. Bio-resource Stress Manag. 2022, 13, 595–604, doi: 10.23910/1.2022.2941b

[38] Khan M.M.H.; Rafii M.Y.; Ramlee S.I.; Jusoh M.; Mamun A. Genetic Variability, Heritability, and Clustering Pattern Exploration of Bambara Groundnut (Vigna subterranea L. Verdc) Accessions for the Perfection of Yield and Yield-Related Traits. Biomed Res. Int. 2020, 2020, 2195797, doi: 10.1155/2020/2195797 33415143 PMC7769641

[39] Terfa G.N.; Gurmu G.N. Genetic Variability, Heritability and Genetic Advance in Linseed (Linum usitatissimum L) Genotypes for Seed Yield and Other Agronomic Traits. Oil Crop Sci. 2020, 5, 156–160, doi: 10.1016/j.ocsci.2020.08.002

[40] Vimal V.K.; Singh P.K.; Pandey V.P. Assess the Genetic Diversity for Growth Yield and Quality Characters among the Genotypes of Turmeric. Plant Arch. 2018, 18, 1026–1032.

[41] Nandakumar K; Fakrudin B; Bn, M.; Venkatesha J; Gk, R. Genetic Variability of Selected Morphological Traits in Turmeric (Curcuma longa L.). Pharma Innov. J. 2022, 874, 874–877.

[42] Singh B., Kumawat P. and Verma A., Studies on Genetic Variability, Hertability and Genetic Advance in Turmeric (Curcuma longa L.). Int. J. Curr. Microbiol. App. Sci., 2018, 7(7), 3169–3176.

[43] Sivakumar V., Chandrasekar R.C., Bhagavan B.V.K. and Ravindrakumar K., Genetic Variability, Heritability and Genetic Advance Studies in Turmeric (Curcuma longa L.) Under High Altitudearea of Andhra Pradesh. Environ. Ecol. 2021, 39, 697–701.

[44] Mamatha K.; Rao M.B.N.; Bhagavan B.; Umajyothi K.; Anuradha M.; Sivaraju K. Studies on Genetic Variability and Heritability in Turmeric (Curuma longa L.). Int. J. Curr. Microbiol. Appl. Sci. 2020, 9, 329–335, doi: 10.20546/ijcmas.2020.909.042

[45] Mukherjee S., Development of a Diagnostic Platform to Detect Protein Biomarkers of Infectious Diseases. Development, 2020, 04–28.

[46] Krishi B.C.; Bengal W.; Krishi B.C.; Bengal W. Genetic Variability, Heritability and Genetic Advance in Turmeric (Curcuma longa L.) Germplasm under Gangetic Alluvial Plains of West Bengal. 2018, 6, 144–146.

[47] Sivakumar V., Umajyothi K., Dorajeerao A.V.D. and Umakrishna K., 2019. Genetic variability, heritability and genetic advance as per cent mean in turmeric (Curcuma longa L.) genotypes. J. Pharmacogn. Phytochem. 2019, 8, 1799–1801.

[48] Singh D.; Mishra D.P.; Pandey V.P.; Kumar M.; Kumar S. Studies on Path Coefficient for Growth and Yield Attributing Traits in Turmeric (Curcuma longa L.). 2021, 10, 2863–2867.

[49] Plant C.-D. Indian Journal of Agriculture and Allied Sciences. Convergence 2016, 2.

[50] Neetu; Dwivedi, S.; Maurya, B.K. Genetic Variability, Heritability and Genetic Advance of Turmeric (Curcuma longa L.) in Bundelkhand Region of India. 2022.

[51] Vijayan, A. Genetic Analysis of Phenological Variations for Yield and Quality in Turmeric (Curcuma longa L.). College of Agriculture Vellayani, Thiruvananthapuram-695 522 Kerala, India, 2015.

[52] Islam M.; Raffi S.; Hossain M.; Hasan A. Analysis of Genetic Variability, Heritability and Genetic Advance for Yield and Yield Associated Traits in Some Promising Advanced Lines of Rice. Progress. Agric. 2015, 26, 26–31, doi: 10.3329/pa.v26i1.24511

[53] Ramachandran; Muthuswami, S. Studies on the Influence of Methods of Planting and Spacing on Yield and Quality of Turmeric. South Indian Hortic. 1984, 32, 85–87.

[54] Pourdad S.S. Repeatability and Relationships among Parametric and Non-Parametric Yield Stability Measures in Safflower (Carthamus tinctorius L.) Genotypes. Crop Breed. J. 2011, 1, 109–118, doi: 10.22092/cbj.2011.100360

[55] Changizi M.; Choukan R.; Heravan E.M.; Bihamta M.R.; Darvish F. Evaluation of Genotype × Environment Interaction and Stability of Corn Hybrids and Relationship among Univariate Parametric Methods. Can. J. Plant Sci. 2014, 94, 1255–1267, doi: 10.4141/CJPS2013-386

[56] Afzal O.; Hassan F.; Ahmed M.; Shabbir G.; Ahmad S. Determination of Stable Safflower Genotypes in Variable Environments by Parametric and Non-Parametric Methods. J. Agric. Food Res. 2021, 6, 100233, doi: 10.1016/j.jafr.2021.100233

[57] Sounderarajan A.; Suresh J.; Prasath D. Variability and Association Analysis of Curcumin Content with Yield Components in Turmeric (Curcuma longa L.). Electron. J. Plant Breed. 2018, 9, 295–303, doi: 10.5958/0975-928X.2018.00034.0

[58] Dudekula M.V.; Kandasamy V.; Balaraman S.S.; Selvamani S.B.; Muthurajan R.; Adhimoolam K.; Manoharan B.; Natesan S. Unlocking the Genetic Diversity of Indian Turmeric (Curcuma longa L.) Germplasm Based on Rhizome Yield Traits and Curcuminoids. Front. Plant Sci. 2022, 13, doi: 10.3389/fpls.2022.1036592 36589076 PMC9797976

[59] Hossain M.A.; Akamine H.; Ishimine Y.; Teruya R.; Aniya Y.; Yamawaki K. Effects of Relative Light Intensity on the Growth, Yield and Curcumin Content of Turmeric (Curcuma longa L.) in Okinawa, Japan. Plant Prod. Sci. 2009, 12, 29–36, doi: 10.1626/pps.12.29

[60] Patel P.; Patel R.; Modha K.; Singh T.J.; Singh M. Path and Correlation Coefficient Analysis for Fourteen Different Morphological Characters in Turmeric (Curcuma longa L.). Int. J. Chem. Stud. 2021, 9, 52–57, doi: 10.22271/chemi.2021.v9.i6a.12130

[61] Singh V.P.; Singh A.K.; Maurya B.P.; Kasera S.; Pandey V.P. Studies on Correlation Coefficient in Turmeric (Curcuma longa L.). Plant Arch. 2018, 18, 97–100.

[62] Netsere A.; Weldemichael G. Non-Destructive Method for Estimating Leaf Area of Turmeric (Curcuma longa). 2013.

[63] Revathy, S.; Andrews, L.D..; P.S., R.; Rajavarman, V.N. An Effective Approach for Turmeric Growth Detection Using Multilevel Linear Algorithm. 2022, doi:10.5281/zenodo.7188915.

[64] Rana S.; Neelam S.; Badiyala D.; Kumar R. Weed Management in Turmeric. Indian J. Weed Sci. 2017, 49, 51–57, doi: 10.5958/0974-8164.2017.00013.2

[65] Singh D.; Kumar R.; Walia S.S.; Brar A. Correlation and Regression Studies of Yield and Yield Components as Influenced by Organic and Inorganic Sources of Nitrogen in Turmeric. 2018, 420–424.

[66] Kumari S.; Singh P.; Singh R.; Khan N. Assessment of Morphological and Biochemical Diversity in Curcuma Longa L. Germplasm by SDS-PAGE. 2017.

[67] Ashraf K.; Ahmad A.; Shah S.A.; Mujeeb M. Genetic Diversity in Accessions of Indian Turmeric (Curcuma Longa L.) Using RAPD Markers. Int. J. Pharm. Pharm. Sci. 2017, 9, 288, doi: 10.22159/ijpps.2017v9i10.18715

[68] Khan S.; Naz S.; Naeem R. Genetic Fingerprinting of Local Turmeric Genotypes Using RAPDs. Pakistan J. Bot. 2013, 45, 339–346.

[69] Bahadur V.; Yeshudas V.; Meena O.P. Nature and Magnitude of Genetic Variability and Diversity Analysis of Indian Turmeric Accessions Using Agro-Morphological Descriptors. Can. J. Plant Sci. 2016, 96, 371–381, doi: 10.1139/cjps-2015-0228

[70] Verma S.; Singh S.; Sharma S.; Tewari S.K.; Roy R.K.; Goel A.K.; et al. Assessment of Genetic Diversity in Indigenous Turmeric (Curcuma Longa) Germplasm from India Using Molecular Markers. Physiol. Mol. Biol. plants an Int. J. Funct. plant Biol. 2015, 21, 233–242, doi: 10.1007/s12298-015-0286-2 25964716 PMC4411392

[71] Singh S.; Panda M.K.; Nayak S. Evaluation of Genetic Diversity in Turmeric (Curcuma Longa L.) Using RAPD and ISSR Markers. Ind. Crops Prod. 2012, 37, 284–291, 10.1016/j.indcrop.2011.12.022

[72] Aswathi A.P.; Raghav S.B.; Prasath D. Assessment of Genetic Variation in Turmeric (Curcuma Longa L.) Varieties Based on Morphological and Molecular Characterization. Genet. Resour. Crop Evol. 2022, 70, 147–158, doi: 10.1007/s10722-022-01417-3

[73] Alam M.A.; Rahman M.; Ahmed S.; Jahan N.; Khan M.A.A.; Islam M.R.; et al. Genetic Variation and Genotype by Environment Interaction for Agronomic Traits in Maize (Zea Mays L.) Hybrids. Plants 2022, 11, doi: 10.3390/plants11111522 35684294 PMC9182618

[74] Sigrist M.S.; Pinheiro J.; Azevedo Filho J.; Zucchi M.I. Genetic Diversity of Turmeric Germplasm (Curcuma Longa; Zingiberaceae) Identified by Microsatellite Markers. Genet. Mol. Res. 2011, 10, 419–428, doi: 10.4238/vol10-1gmr1047 21425092

[75] Tavares K.; Kirk E.; Motomura-Wages S.; Calpito J.; Bingham J.-P.; Ahmad A.A.; et al. Genotypic and Environmental Influence on Fresh Rhizome Yield of Turmeric (Curcuma Longa L.). Agronomy 2022, 12.

