# Supplementary material for: Study on the genetic variability and adaptability of turmeric (Curcuma longa L.) genotypes for development of desirable cultivars
Source: PLoS One. 2024 Jan 19;19(1):e0297202. doi: 10.1371/journal.pone.0297202 (PMC10798502; doi:10.1371/journal.pone.0297202)
Supplement: S1 Table — (DOCX) [file pone.0297202.s001.docx]

**Table S1**. List of genotypes with local name and source used in the present study

| **GEN** | **Local name** | **Source** |
| --- | --- | --- |
| BARI Holud-1 | Dimla | Bangladesh Agricultural Research Institute (BARI) |
| BARI Holud-2 | Sinduri | BARI |
| BARI Holud-3 | - | BARI |
| BARI Holud-4 | - | BARI |
| BARI Holud-5 | - | BARI |
| T0008 | - | BARI |
| T0012 | - | BARI |
| T0013 | - | BARI |
| T0015 | - | BARI |
| T0016 | - | BARI |
| T0017 | - | BARI |
| T0019 | - | BARI |
| T0023 | - | BARI |
| T0052 | - | BARI |
| T0061 | - | BARI |
| T0063 | - | BARI |
| T0066 | - | BARI |
| T0077 | - | BARI |
| T0082 | Harinpilo | Ishurdi, Pabna |
| T0083 | Arani | Ishurdi, Pabna |
| T0084 | Gaita | Ishurdi, Pabna |
| T0085 | - | Khagrachhari |
| T0093 | Lofa | Hatgopalpur |
| T0094 | Adarpana | Shailokupa |
| T0095 | Adagaita | Magura |
| T0095-1 | - | Magura |
| T0096 | Harinpilo | Ilishkholi, Baliakandi, Rajbari |
| T0097 | - | Thailand |
| T0098 | - | Uthuli, Shibganj, Bogura |
| T0102 | Guimara | Guimara, Khagrachhari |
| T0103 | Matiranga | Matiranga, Khagrachhari |
| T0104 | Tholibari | Khagrachhari |
| T0105 | Patnai | Tetulia, Panchagarh |
| T0106 | - | Khagrachhari Sadar |
| T0107 | - | Dighinal, Khagrachhari |
| T0108 | - | Batchhari, Rangamati |
| T0109 | - | Betchhari, Rangamati |
| T0116 | Joy-1 | Joydebpur, Gazipur |
| T0117 | Joy-5 | Joydebpur, Gazipur |
| T0118 | Makel Bare | Kapilmuni, Magura |
| T0119 | Patnai | Jhaodia, Kushtia |
| T0121 | Thailand | Bowangchhari, Bandarban |
| T0122 | - | Dagnachhari, Bandarban |
| T0123 | - | Ramangchhari, Bandarban |
| T0124 | - | Dagnachhari, Bandarban |
| T0126 | K5 | Habiganj |
| T0127 | Chewing | Bandarban |
| T0128 | - | Ghatail, Tangail |
| T0129 | - | Baroghoriapara, Debiganj, Panchagarh |
| T0130 | Surjamukhi | Baroghoriapara, Debiganj, Panchagarh |
| T0132 | - | Rajsthali, Rangamati |
| T0133 | - | Kaokhali, Rangamati |
| T0134 | - | Rajsthali, Rangamati |
