# Supplementary material for: Study on the genetic variability and adaptability of turmeric (Curcuma longa L.) genotypes for development of desirable cultivars
Source: PLoS One. 2024 Jan 19;19(1):e0297202. doi: 10.1371/journal.pone.0297202 (PMC10798502; doi:10.1371/journal.pone.0297202)
Supplement: S2 Table — (DOCX) [file pone.0297202.s002.docx]

**Table S2.** Prevailed weather condition during the study at the experimental site during 2019-2020, 2020-2021 and 2021-2022

| **Season** | **Month** | **T_Mean (°C)** | **T_Max (°C)** | **T_Min (°C)** | **Rainfall (mm)** | **RH (%)** |
| --- | --- | --- | --- | --- | --- | --- |
| 2019-20 | APR | 27.65 | 37.83 | 19.78 | 247.85 | 64.81 |
|  | MAY | 30.84 | 40.87 | 21.73 | 263.67 | 69.06 |
|  | JUN | 29.69 | 35.15 | 25.97 | 490.43 | 85.62 |
|  | JUL | 28.85 | 33.01 | 26.02 | 727.73 | 90.12 |
|  | AUG | 29.13 | 34.26 | 25.77 | 263.67 | 87.19 |
|  | SEP | 28.02 | 33.25 | 22.77 | 485.16 | 87.81 |
|  | OCT | 25.62 | 30.83 | 18.8 | 305.86 | 85.44 |
|  | NOV | 22.67 | 28.85 | 15.05 | 10.55 | 84.19 |
|  | DEC | 16.74 | 25.73 | 5.76 | 5.27 | 81.94 |
|  | JAN | 15.88 | 25.11 | 7.44 | 26.37 | 77.88 |
|  | FEB | 18.59 | 28.8 | 7.26 | 0 | 64.56 |
|  | MAR | 24.73 | 36.4 | 14.92 | 52.73 | 57.31 |
| 2020-21 | APR | 30.58 | 40.08 | 19.12 | 69.13 | 45.81 |
|  | MAY | 28.23 | 34.54 | 22.81 | 585.35 | 80.75 |
|  | JUN | 28.74 | 32.55 | 25.62 | 485.16 | 89.06 |
|  | JUL | 28.75 | 32.74 | 25.65 | 495.7 | 90.25 |
|  | AUG | 28.91 | 34.17 | 25.01 | 274.22 | 88.12 |
|  | SEP | 28.38 | 32.87 | 25.62 | 421.88 | 89.81 |
|  | OCT | 27.64 | 32.76 | 20.86 | 326.95 | 83.94 |
|  | NOV | 21.83 | 30.14 | 11.87 | 0 | 83 |
|  | DEC | 17.66 | 26.11 | 7.85 | 0 | 78.88 |
|  | JAN | 17.12 | 26.31 | 6.73 | 0 | 71.31 |
|  | FEB | 20.79 | 33.57 | 7.94 | 0 | 52.81 |
|  | MAR | 27.5 | 38.61 | 15.69 | 5.27 | 41.06 |
| 2021-22 | APR | 31.22 | 38.05 | 24.95 | 15.17 | 53.25 |
|  | MAY | 29.15 | 38.05 | 23.53 | 365.47 | 74.06 |
|  | JUN | 28.78 | 34.94 | 24.87 | 405.78 | 86.5 |
|  | JUL | 29.01 | 34.06 | 25.48 | 289.38 | 86.81 |
|  | AUG | 28.48 | 34.4 | 25.37 | 449.95 | 89.19 |
|  | SEP | 28.04 | 33.01 | 24.42 | 196.07 | 87.56 |
|  | OCT | 26.53 | 34.14 | 17.39 | 246.24 | 85.75 |
|  | NOV | 21.03 | 27.11 | 13.62 | 3.11 | 81.19 |
|  | DEC | 18.1 | 26.43 | 6.58 | 11.27 | 80.94 |
|  | JAN | 16.76 | 22.39 | 11.92 | 24.82 | 79.04 |
|  | FEB | 18.14 | 24.04 | 12.32 | 53.46 | 69.91 |
|  | MAR | 26.40 | 34.02 | 19.26 | 4.22 | 52.64 |

T_max=Maximum temperature; T_min=Minimum temperature; T_Mean=Mean Temperature; RH=Relative humidity
