# Supplementary material for: Study on the genetic variability and adaptability of turmeric (Curcuma longa L.) genotypes for development of desirable cultivars
Source: PLoS One. 2024 Jan 19;19(1):e0297202. doi: 10.1371/journal.pone.0297202 (PMC10798502; doi:10.1371/journal.pone.0297202)
Supplement: S3 Table — (DOCX) [file pone.0297202.s003.docx]

**Table S3**. Physical properties of initial soil of the experimental plot, 2019-2020, 2020-2021 and 2021-2022

| **Particle size distribution** | **Year 1** | **Year 2** | **Year 3** |
| --- | --- | --- | --- |
| Sand (%) | 63.28 | 61.58 | 64.21 |
| Silt (%) | 26.67 | 26.51 | 25.10 |
| Clay (%) | 10.05 | 11.91 | 10.69 |
| Textural class | Sandy Loam | Sandy Loam | Sandy Loam |
| Bulk density (g/cm^3^) | 1.41 | 1.45 | 1.43 |
| Particle density (g/cm^3^) | 2.21 | 2.25 | 2.23 |
| Total porosity (%) | 36.20 | 35.56 | 35.87 |
| Field capacity (%) | 29.66 | 31.27 | 30.73 |
