# Supplementary material for: Study on the genetic variability and adaptability of turmeric (Curcuma longa L.) genotypes for development of desirable cultivars
Source: PLoS One. 2024 Jan 19;19(1):e0297202. doi: 10.1371/journal.pone.0297202 (PMC10798502; doi:10.1371/journal.pone.0297202)
Supplement: S4 Table — (DOCX) [file pone.0297202.s004.docx]

**Table S4.** Chemical properties of initial and post-harvest soil of the experimental site, 2019-2020, 2020-2021 and 2021-2022

| **Soil characteristics** | **Analytical value**  **(Initial soil)** | | | **Analytical value**  **(Soil after harvest)** | | | **Critical levels** |
| --- | --- | --- | --- | --- | --- | --- | --- |
|  | **Year 1** | **Year 2** | **Year 3** | **Year 1** | **Year 2** | **Year 3** |  |
| Soil p^H^ | 6.00 | 6.23 | 6.75 | 7.10 | 7.25 | 6.91 | - |
| Organic matter (%) | 1.33 | 1.39 | 1.35 | 1.10 | 1.25 | 1.18 | C:N= 10:1 |
| Total N (%) | 0.07 | 0.09 | 0.07 | 0.05 | 0.08 | 0.09 | 0.12 |
| Available P (µg/g soil) | 30.77 | 28.21 | 29.75 | 37.33 | 31.25 | 35.62 | 10.0 |
| Exchangeable K (meq/100g soil) | 0.15 | 0.17 | 0.19 | 0.27 | 0.21 | 0.25 | 0.12 |
| Available S (µg/g soil) | 10.57 | 11.37 | 12.28 | 20.33 | 23.31 | 25.41 | 10.0 |
| Available Zn (µg/g soil) | 1.63 | 1.55 | 1.71 | 1.57 | 1.65 | 1.53 | 0.6 |
| Available Boron (µg/g soil) | 0.20 | 0.25 | 0.27 | 0.13 | 0.15 | 0.17 | 0.2 |
| Available Cu (µg/g soil) | 2.31 | 2.51 | 2.23 | 2.20 | 2.34 | 2.39 | 0.2 |
| Available Fe (µg/g soil) | 55.91 | 57.25 | 55.89 | 51.30 | 41.21 | 45.58 | 4.0 |
| Available Mn (µg/g soil) | 18.21 | 15.24 | 19.25 | 16.47 | 15.51 | 17.55 | 1.0 |
| Exchangeable Ca (meq/100g soil) | 4.70 | 4.28 | 3.69 | 4.15 | 3.75 | 3.29 | 2.0 |
| Exchangeable Mg (meq/100g soil) | 1.59 | 1.65 | 1.41 | 1.46 | 1.49 | 1.53 | 0.5 |
