# Supplementary material for: Study on the genetic variability and adaptability of turmeric (Curcuma longa L.) genotypes for development of desirable cultivars
Source: PLoS One. 2024 Jan 19;19(1):e0297202. doi: 10.1371/journal.pone.0297202 (PMC10798502; doi:10.1371/journal.pone.0297202)
Supplement: S5 Table — (DOCX) [file pone.0297202.s005.docx]

**Table S5.** Mean performance of 53 genotypes of turmeric grown during the year of 2019-22

| **Genotypes** | **PH** | **NB** | **NL** | **NMR** | **WMR** | **NPF** | **WPF** | **NSF** | **WSF** | **LMR** | **YPP** | **FY** |
| --- | --- | --- | --- | --- | --- | --- | --- | --- | --- | --- | --- | --- |
| BARI Holud-1 | 90.96 | 3.33 | 20.27 | 1.92 | 57.42 | 4.75 | 108.42 | 8.92 | 236.09 | 6.91 | 358.25 | 17.87 |
| BARI Holud-2 | 92.00 | 3.27 | 22.73 | 1.42 | 60.58 | 4.25 | 78.25 | 8.67 | 88.33 | 7.47 | 232.49 | 15.19 |
| BARI Holud-3 | 98.47 | 3.53 | 20.90 | 1.75 | 91.59 | 4.17 | 87.08 | 8.67 | 95.58 | 6.86 | 284.50 | 19.90 |
| BARI Holud-4 | 105.33 | 3.73 | 22.93 | 1.50 | 102.75 | 5.08 | 112.84 | 10.59 | 129.75 | 8.14 | 332.17 | 22.59 |
| BARI Holud-5 | 103.40 | 4.07 | 25.53 | 1.50 | 61.92 | 4.17 | 75.42 | 9.09 | 80.17 | 6.78 | 280.59 | 16.76 |
| T0008 | 101.60 | 3.27 | 20.80 | 1.34 | 115.67 | 4.75 | 120.58 | 12.08 | 207.42 | 7.68 | 506.17 | 14.65 |
| T0012 | 89.73 | 3.27 | 20.20 | 1.59 | 75.75 | 5.00 | 102.17 | 11.67 | 99.59 | 6.97 | 328.50 | 10.94 |
| T0013 | 84.73 | 4.27 | 21.67 | 1.42 | 58.75 | 4.92 | 87.58 | 10.92 | 78.17 | 7.59 | 253.92 | 7.67 |
| T0015 | 101.87 | 4.47 | 24.27 | 1.33 | 89.92 | 5.34 | 92.75 | 8.92 | 88.84 | 8.10 | 269.92 | 28.04 |
| T0016 | 91.27 | 3.13 | 23.47 | 1.84 | 54.34 | 4.34 | 76.33 | 11.42 | 162.67 | 6.34 | 349.59 | 14.86 |
| T0017 | 102.80 | 3.80 | 27.93 | 1.00 | 65.67 | 4.83 | 71.92 | 10.58 | 124.58 | 8.54 | 239.33 | 13.79 |
| T0019 | 86.47 | 4.53 | 24.67 | 1.58 | 97.08 | 6.17 | 91.92 | 12.08 | 66.08 | 7.00 | 415.84 | 22.16 |
| T0023 | 96.40 | 2.40 | 23.73 | 1.17 | 81.50 | 3.42 | 71.58 | 10.33 | 132.50 | 8.15 | 413.17 | 13.15 |
| T0052 | 84.00 | 2.80 | 20.40 | 1.58 | 54.00 | 2.50 | 48.83 | 5.17 | 71.75 | 6.02 | 194.33 | 6.60 |
| T0061 | 103.47 | 3.73 | 22.20 | 1.50 | 118.25 | 4.92 | 111.17 | 7.83 | 94.33 | 7.78 | 361.17 | 26.79 |
| T0063 | 100.93 | 5.00 | 24.27 | 1.17 | 101.92 | 4.59 | 88.17 | 8.50 | 93.08 | 7.74 | 272.34 | 15.18 |
| T0066 | 97.67 | 3.47 | 20.93 | 1.34 | 107.50 | 5.17 | 113.42 | 8.84 | 112.50 | 7.12 | 410.25 | 21.50 |
| T0077 | 81.67 | 3.47 | 18.80 | 2.00 | 59.17 | 4.00 | 103.83 | 6.67 | 154.00 | 5.28 | 293.67 | 10.65 |
| T0082 | 107.07 | 4.80 | 26.80 | 1.75 | 145.42 | 4.42 | 113.58 | 13.67 | 145.92 | 8.55 | 486.50 | 24.57 |
| T0083 | 96.20 | 4.40 | 26.87 | 1.42 | 63.58 | 4.34 | 56.08 | 8.42 | 66.58 | 6.98 | 278.67 | 17.64 |
| T0084 | 103.20 | 3.67 | 24.53 | 1.83 | 85.08 | 5.25 | 102.25 | 9.42 | 110.50 | 7.60 | 332.59 | 20.48 |
| T0085 | 101.87 | 3.87 | 25.53 | 1.59 | 109.92 | 5.25 | 110.25 | 10.50 | 112.33 | 8.04 | 504.92 | 22.32 |
| T0093 | 93.93 | 3.87 | 28.33 | 1.08 | 82.83 | 5.75 | 104.25 | 9.50 | 101.84 | 9.03 | 383.08 | 16.82 |
| T0094 | 108.27 | 3.67 | 26.40 | 2.17 | 111.67 | 5.92 | 87.67 | 8.59 | 105.50 | 7.43 | 357.75 | 25.84 |
| T0095 | 94.07 | 3.40 | 24.47 | 1.50 | 42.92 | 5.50 | 52.75 | 10.25 | 102.33 | 7.02 | 266.25 | 10.55 |
| T0095-1 | 97.27 | 3.93 | 20.67 | 1.42 | 94.67 | 3.58 | 95.67 | 8.17 | 127.58 | 6.98 | 367.75 | 14.18 |
| T0096 | 105.63 | 3.47 | 18.47 | 1.17 | 98.79 | 5.50 | 70.27 | 7.00 | 84.88 | 8.33 | 246.27 | 20.05 |
| T0097 | 106.23 | 3.33 | 22.94 | 1.58 | 111.97 | 5.25 | 101.77 | 10.51 | 135.67 | 7.74 | 361.89 | 18.54 |
| T0098 | 101.23 | 3.47 | 22.31 | 1.42 | 83.54 | 6.25 | 94.03 | 8.75 | 84.21 | 7.49 | 273.48 | 12.15 |
| T0102 | 103.03 | 4.33 | 20.47 | 1.42 | 80.70 | 4.84 | 82.04 | 9.51 | 108.40 | 7.46 | 269.28 | 21.50 |
| T0103 | 113.36 | 5.80 | 30.81 | 1.50 | 106.21 | 5.17 | 170.07 | 8.42 | 97.47 | 7.97 | 433.09 | 27.30 |
| T0104 | 88.70 | 3.20 | 21.01 | 1.42 | 86.31 | 3.17 | 68.78 | 8.67 | 77.64 | 8.03 | 268.48 | 7.96 |
| T0105 | 95.03 | 3.34 | 22.54 | 1.58 | 95.05 | 5.09 | 78.36 | 10.34 | 137.33 | 7.71 | 377.84 | 13.07 |
| T0106 | 109.37 | 4.54 | 28.47 | 1.84 | 128.07 | 5.42 | 94.86 | 10.34 | 136.32 | 7.45 | 519.28 | 24.91 |
| T0107 | 98.50 | 3.67 | 25.40 | 1.42 | 81.00 | 5.83 | 96.17 | 11.92 | 56.50 | 7.65 | 214.25 | 15.30 |
| T0108 | 81.96 | 3.20 | 18.81 | 1.42 | 54.02 | 5.75 | 132.44 | 6.67 | 109.56 | 7.60 | 334.68 | 17.64 |
| T0109 | 91.96 | 3.07 | 19.94 | 1.08 | 35.68 | 2.50 | 38.27 | 5.59 | 56.04 | 6.26 | 120.46 | 4.47 |
| T0116 | 101.23 | 4.27 | 26.21 | 1.50 | 104.57 | 3.83 | 71.87 | 8.76 | 133.60 | 7.25 | 421.44 | 19.96 |
| T0117 | 116.50 | 4.07 | 24.74 | 2.00 | 151.26 | 5.00 | 110.95 | 11.09 | 134.92 | 8.58 | 413.43 | 26.64 |
| T0118 | 106.50 | 3.73 | 25.28 | 1.17 | 75.61 | 3.34 | 122.29 | 8.34 | 130.31 | 6.95 | 342.06 | 19.73 |
| T0119 | 105.55 | 3.73 | 23.20 | 1.67 | 93.18 | 4.75 | 91.76 | 9.09 | 94.44 | 7.88 | 265.55 | 18.89 |
| T0121 | 110.68 | 3.53 | 26.14 | 2.25 | 142.94 | 7.25 | 140.52 | 11.84 | 149.95 | 7.62 | 743.55 | 23.96 |
| T0122 | 81.81 | 3.34 | 20.47 | 1.08 | 30.92 | 3.33 | 55.26 | 8.17 | 92.69 | 5.88 | 258.22 | 10.86 |
| T0123 | 103.95 | 3.00 | 21.07 | 1.59 | 111.02 | 4.83 | 68.10 | 8.25 | 103.78 | 7.54 | 347.58 | 18.45 |
| T0124 | 99.41 | 3.60 | 19.94 | 1.25 | 90.10 | 4.84 | 73.68 | 7.67 | 88.02 | 8.87 | 274.15 | 10.37 |
| T0126 | 89.28 | 3.60 | 21.14 | 1.42 | 116.86 | 3.67 | 129.36 | 12.25 | 197.28 | 7.71 | 465.59 | 17.45 |
| T0127 | 71.13 | 3.33 | 17.60 | 1.42 | 27.59 | 3.92 | 45.50 | 5.92 | 38.92 | 6.01 | 148.00 | 4.00 |
| T0128 | 95.72 | 3.70 | 19.94 | 1.50 | 84.10 | 4.50 | 79.85 | 10.00 | 94.36 | 7.29 | 267.57 | 13.42 |
| T0129 | 116.28 | 3.53 | 25.20 | 1.67 | 112.28 | 4.67 | 107.94 | 9.58 | 152.71 | 7.12 | 450.36 | 25.75 |
| T0130 | 89.15 | 2.67 | 19.27 | 1.50 | 48.51 | 3.00 | 41.42 | 6.67 | 68.19 | 7.09 | 190.55 | 8.64 |
| T0132 | 99.68 | 3.67 | 27.00 | 1.83 | 154.10 | 6.92 | 154.51 | 12.00 | 127.94 | 8.04 | 468.65 | 17.02 |
| T0133 | 93.41 | 3.20 | 19.94 | 1.42 | 64.01 | 4.33 | 63.51 | 7.75 | 72.94 | 7.48 | 208.96 | 12.31 |
| T0134 | 92.28 | 3.73 | 24.21 | 1.75 | 101.76 | 4.92 | 111.26 | 11.00 | 107.68 | 8.12 | 418.12 | 11.91 |

PH=Plant Height; NB=Number of branch; NL=Number of leaves; NMR=Number of mother rhizome; WMR=Weight of mother rhizome; NPF=Number of primary finger; WPF=Weight of primary finger; NSF=Number of secondary finger; WSF=Weight of secondary finger; MRL=Length of mother rhizome; YPP= Yield per plant; FY=Fresh yield;.
