# Supplementary material for: Study on the genetic variability and adaptability of turmeric (Curcuma longa L.) genotypes for development of desirable cultivars
Source: PLoS One. 2024 Jan 19;19(1):e0297202. doi: 10.1371/journal.pone.0297202 (PMC10798502; doi:10.1371/journal.pone.0297202)
Supplement: S6 Table — (DOCX) [file pone.0297202.s006.docx]

**Table S6**. Mean performance of 53 genotypes of turmeric grown during the year of 2019-20

| **Sl. No.** | **Genotype** | **PH** | **NB** | **NL** | **NMR** | **WMR** | **NPF** | **WPF** | **NSF** | **WSF** | **LMR** | **YPP** | **FY** |
| --- | --- | --- | --- | --- | --- | --- | --- | --- | --- | --- | --- | --- | --- |
| 1 | BARI Holud-1 | 85.40 | 2.00 | 15.80 | 2.50 | 37.00 | 5.51 | 50.51 | 5.51 | 22.75 | 4.91 | 140.00 | 9.50 |
| 2 | BARI Holud-2 | 63.00 | 1.80 | 14.00 | 1.25 | 32.50 | 4.50 | 25.75 | 8.25 | 35.00 | 6.60 | 67.00 | 3.20 |
| 3 | BARI Holud-3 | 82.20 | 2.20 | 15.40 | 1.75 | 49.75 | 4.00 | 69.00 | 9.50 | 27.25 | 5.32 | 134.00 | 6.10 |
| 4 | BARI Holud-4 | 115.00 | 2.40 | 21.60 | 1.76 | 119.25 | 7.50 | 122.75 | 9.00 | 35.75 | 9.15 | 224.00 | 17.90 |
| 5 | BARI Holud-5 | 85.40 | 2.00 | 18.40 | 1.75 | 27.50 | 5.25 | 54.00 | 7.25 | 26.50 | 4.75 | 131.00 | 8.65 |
| 6 | T0008 | 75.60 | 2.20 | 14.60 | 1.00 | 42.75 | 4.25 | 40.25 | 5.50 | 20.50 | 6.50 | 90.00 | 4.32 |
| 7 | T0012 | 81.60 | 2.00 | 15.20 | 1.50 | 59.25 | 5.50 | 78.00 | 9.75 | 31.25 | 4.98 | 153.00 | 9.20 |
| 8 | T0013 | 58.20 | 1.60 | 12.20 | 1.75 | 34.00 | 6.50 | 52.00 | 13.50 | 41.25 | 6.32 | 118.00 | 4.51 |
| 9 | T0015 | 100.00 | 2.60 | 19.80 | 1.75 | 57.25 | 9.50 | 56.00 | 11.51 | 28.51 | 5.99 | 211.00 | 10.40 |
| 10 | T0016 | 82.80 | 2.40 | 21.40 | 2.51 | 45.75 | 6.75 | 75.75 | 11.51 | 51.25 | 4.39 | 329.00 | 11.60 |
| 11 | T0017 | 83.00 | 1.80 | 15.00 | 1.00 | 48.00 | 5.50 | 61.75 | 9.25 | 34.50 | 8.43 | 160.00 | 8.60 |
| 12 | T0019 | 79.20 | 3.00 | 21.40 | 2.51 | 83.00 | 7.75 | 110.51 | 14.25 | 35.75 | 5.69 | 277.00 | 17.72 |
| 13 | T0023 | 90.20 | 2.00 | 17.60 | 1.25 | 45.00 | 7.25 | 98.00 | 10.00 | 49.75 | 6.08 | 230.00 | 12.23 |
| 14 | T0052 | 61.00 | 1.40 | 15.40 | 1.51 | 31.50 | 2.75 | 36.75 | 3.00 | 13.00 | 3.80 | 78.00 | 2.24 |
| 15 | T0061 | 99.40 | 3.00 | 18.00 | 1.75 | 77.25 | 5.51 | 67.00 | 8.75 | 25.25 | 5.91 | 271.00 | 10.11 |
| 16 | T0063 | 91.00 | 2.20 | 15.80 | 1.00 | 35.25 | 4.51 | 42.51 | 5.25 | 19.00 | 6.51 | 153.00 | 5.30 |
| 17 | T0066 | 100.60 | 3.20 | 17.40 | 1.51 | 58.00 | 3.51 | 44.75 | 9.50 | 36.25 | 4.77 | 284.00 | 10.20 |
| 18 | T0077 | 57.20 | 1.80 | 11.00 | 1.25 | 13.51 | 4.00 | 35.75 | 3.00 | 10.51 | 3.60 | 128.00 | 1.50 |
| 19 | T0082 | 101.40 | 2.60 | 18.20 | 1.50 | 79.25 | 5.25 | 71.00 | 15.26 | 52.50 | 7.26 | 389.00 | 20.40 |
| 20 | T0083 | 74.80 | 2.60 | 18.80 | 1.25 | 33.75 | 5.00 | 25.75 | 6.51 | 19.25 | 5.23 | 84.00 | 1.90 |
| 21 | T0084 | 96.60 | 3.40 | 29.40 | 2.00 | 43.75 | 6.50 | 82.25 | 8.00 | 36.75 | 7.59 | 123.00 | 16.00 |
| 22 | T0085 | 111.20 | 3.20 | 28.40 | 2.26 | 171.26 | 7.50 | 158.00 | 10.76 | 92.50 | 8.34 | 499.00 | 25.00 |
| 23 | T0093 | 87.80 | 4.20 | 29.00 | 1.00 | 38.25 | 6.50 | 55.00 | 5.25 | 10.51 | 6.28 | 204.00 | 7.00 |
| 24 | T0094 | 101.80 | 3.60 | 25.60 | 2.50 | 98.76 | 8.75 | 80.76 | 9.76 | 34.76 | 6.47 | 307.00 | 25.30 |
| 25 | T0095 | 84.00 | 2.80 | 20.20 | 1.75 | 48.00 | 5.25 | 64.00 | 8.00 | 60.75 | 4.74 | 241.00 | 7.40 |
| 26 | T0095-1 | 74.60 | 2.20 | 13.60 | 1.25 | 24.51 | 3.50 | 21.50 | 3.75 | 12.00 | 4.93 | 112.00 | 2.10 |
| 27 | T0096 | 96.00 | 2.60 | 19.40 | 1.51 | 96.00 | 10.25 | 63.00 | 7.75 | 76.51 | 6.48 | 214.00 | 10.20 |
| 28 | T0097 | 94.80 | 2.80 | 19.60 | 1.50 | 49.00 | 5.50 | 52.75 | 7.75 | 21.50 | 5.20 | 214.00 | 7.50 |
| 29 | T0098 | 94.40 | 2.80 | 16.40 | 1.75 | 41.51 | 7.25 | 65.25 | 11.25 | 35.51 | 4.78 | 339.00 | 9.64 |
| 30 | T0102 | 80.80 | 3.00 | 17.00 | 1.75 | 69.00 | 5.51 | 60.75 | 7.51 | 42.51 | 6.68 | 238.00 | 9.80 |
| 31 | T0103 | 127.60 | 3.60 | 28.40 | 2.00 | 141.50 | 8.25 | 154.00 | 9.50 | 64.25 | 7.26 | 542.00 | 18.00 |
| 32 | T0104 | 74.20 | 2.60 | 14.80 | 1.00 | 18.75 | 3.50 | 27.75 | 3.00 | 10.00 | 6.35 | 154.00 | 2.20 |
| 33 | T0105 | 82.20 | 2.20 | 16.20 | 2.00 | 81.75 | 9.00 | 69.25 | 9.50 | 98.75 | 6.52 | 404.00 | 12.20 |
| 34 | T0106 | 115.40 | 3.20 | 25.60 | 1.76 | 108.50 | 7.50 | 109.50 | 10.26 | 67.26 | 7.93 | 698.00 | 28.00 |
| 35 | T0107 | 106.60 | 2.80 | 23.00 | 1.00 | 56.51 | 3.75 | 44.51 | 13.51 | 30.75 | 7.08 | 82.00 | 11.60 |
| 36 | T0108 | 59.40 | 2.20 | 15.40 | 2.00 | 52.00 | 8.25 | 141.00 | 7.25 | 49.75 | 6.20 | 347.00 | 13.71 |
| 37 | T0109 | 63.20 | 2.00 | 12.60 | 1.00 | 22.25 | 2.75 | 23.50 | 4.00 | 17.00 | 3.88 | 49.00 | 1.60 |
| 38 | T0116 | 89.40 | 3.20 | 24.20 | 1.00 | 50.50 | 4.00 | 28.75 | 5.75 | 19.00 | 6.75 | 206.00 | 7.20 |
| 39 | T0117 | 124.00 | 2.40 | 21.00 | 1.76 | 99.75 | 7.25 | 96.50 | 14.50 | 74.75 | 8.09 | 421.00 | 26.06 |
| 40 | T0118 | 90.80 | 2.60 | 18.40 | 1.25 | 35.00 | 3.51 | 38.00 | 8.25 | 34.51 | 5.68 | 120.00 | 10.00 |
| 41 | T0119 | 97.00 | 2.20 | 16.40 | 1.75 | 78.75 | 5.51 | 61.00 | 8.75 | 29.25 | 6.60 | 160.00 | 16.31 |
| 42 | T0121 | 117.00 | 3.00 | 27.40 | 2.00 | 122.76 | 9.50 | 150.26 | 13.76 | 108.00 | 5.59 | 1458.00 | 22.00 |
| 43 | T0122 | 59.40 | 3.20 | 17.60 | 1.00 | 9.75 | 2.00 | 18.51 | 3.25 | 9.25 | 4.10 | 166.00 | 2.30 |
| 44 | T0123 | 82.40 | 1.80 | 12.80 | 2.00 | 94.00 | 5.50 | 37.75 | 6.00 | 17.50 | 6.88 | 290.00 | 7.60 |
| 45 | T0124 | 70.40 | 2.00 | 12.80 | 1.25 | 40.51 | 6.00 | 66.51 | 7.51 | 31.25 | 5.43 | 74.00 | 4.34 |
| 46 | T0126 | 83.00 | 2.00 | 14.60 | 1.75 | 61.25 | 4.75 | 47.51 | 10.75 | 27.51 | 5.03 | 108.00 | 2.56 |
| 47 | T0127 | 65.00 | 2.20 | 15.20 | 1.00 | 14.75 | 3.00 | 18.00 | 4.25 | 13.00 | 5.80 | 69.00 | 3.50 |
| 48 | T0128 | 75.00 | 2.00 | 13.40 | 2.00 | 57.00 | 6.00 | 43.00 | 9.00 | 35.25 | 5.31 | 104.00 | 2.30 |
| 49 | T0129 | 120.40 | 2.80 | 25.60 | 1.50 | 70.76 | 5.76 | 75.26 | 8.00 | 37.50 | 7.16 | 276.00 | 20.74 |
| 50 | T0130 | 69.00 | 1.40 | 11.40 | 1.25 | 22.75 | 3.25 | 27.00 | 5.00 | 14.50 | 5.71 | 85.00 | 3.10 |
| 51 | T0132 | 86.40 | 3.20 | 25.40 | 1.25 | 62.75 | 4.00 | 93.00 | 11.51 | 91.75 | 6.36 | 262.00 | 10.67 |
| 52 | T0133 | 81.00 | 2.40 | 15.60 | 2.00 | 48.00 | 6.00 | 42.25 | 4.75 | 20.25 | 6.20 | 135.00 | 5.72 |
| 53 | T0134 | 79.80 | 3.20 | 23.20 | 2.51 | 83.00 | 4.51 | 72.75 | 10.25 | 74.00 | 6.40 | 501.00 | 5.32 |

PH= Plant Height; NB= Number of branches; NL= Number of leaves; NMR= Number of mother rhizome; WMR= Weight of mother rhizome; NPF= Number of primary fingers; WPF= Weight of primary finger; NSF= Number of secondary fingers; WSF= Weight of secondary finger; MRL= Length of mother rhizome; YPP= Yield per plant; FY= Fresh yield;
