# Supplementary material for: Study on the genetic variability and adaptability of turmeric (Curcuma longa L.) genotypes for development of desirable cultivars
Source: PLoS One. 2024 Jan 19;19(1):e0297202. doi: 10.1371/journal.pone.0297202 (PMC10798502; doi:10.1371/journal.pone.0297202)
Supplement: S7 Table — (DOCX) [file pone.0297202.s007.docx]

**Table S7.** Mean performance of 53 genotypes of turmeric grown during the year of 2020-21

| **Sl. No.** | **Genotype** | **PH** | **NB** | **NL** | **NMR** | **WMR** | **NPF** | **WPF** | **NSF** | **WSF** | **LMR** | **YPP** | **FY** |
| --- | --- | --- | --- | --- | --- | --- | --- | --- | --- | --- | --- | --- | --- |
| 1 | BARI Holud-1 | 97.80 | 2.40 | 24.20 | 1.75 | 103.00 | 1.75 | 83.25 | 12.25 | 500.50 | 8.87 | 526.00 | 27.10 |
| 2 | BARI Holud-2 | 105.60 | 3.00 | 28.20 | 1.25 | 65.25 | 2.00 | 75.25 | 8.51 | 129.00 | 8.25 | 311.71 | 29.76 |
| 3 | BARI Holud-3 | 95.60 | 2.80 | 23.31 | 2.00 | 115.51 | 1.75 | 70.75 | 8.00 | 152.00 | 8.34 | 381.00 | 18.60 |
| 4 | BARI Holud-4 | 96.60 | 2.80 | 24.40 | 1.25 | 58.25 | 1.75 | 51.51 | 10.51 | 195.51 | 8.07 | 319.51 | 10.58 |
| 5 | BARI Holud-5 | 100.60 | 2.80 | 28.20 | 1.75 | 106.00 | 1.75 | 68.51 | 14.25 | 172.75 | 7.94 | 512.51 | 17.62 |
| 6 | T0008 | 101.60 | 2.60 | 22.20 | 1.75 | 195.00 | 2.75 | 189.00 | 21.25 | 504.00 | 8.51 | 1106.50 | 17.94 |
| 7 | T0012 | 98.20 | 2.60 | 22.40 | 1.51 | 102.75 | 2.50 | 105.00 | 14.25 | 193.25 | 7.88 | 569.51 | 12.63 |
| 8 | T0013 | 99.00 | 2.80 | 24.60 | 1.00 | 39.75 | 1.75 | 77.75 | 10.51 | 114.51 | 8.75 | 329.51 | 12.21 |
| 9 | T0015 | 97.40 | 3.00 | 26.40 | 1.25 | 113.25 | 1.25 | 67.51 | 9.25 | 158.51 | 9.50 | 284.00 | 28.71 |
| 10 | T0016 | 101.80 | 2.60 | 30.40 | 1.75 | 64.00 | 2.51 | 83.25 | 10.25 | 232.00 | 7.75 | 394.00 | 15.48 |
| 11 | T0017 | 100.20 | 3.00 | 33.20 | 1.00 | 114.00 | 1.75 | 77.75 | 12.25 | 248.00 | 10.07 | 353.00 | 22.78 |
| 12 | T0019 | 87.80 | 3.40 | 26.60 | 1.25 | 168.25 | 3.25 | 99.51 | 15.75 | 103.25 | 9.88 | 805.00 | 34.76 |
| 13 | T0023 | 98.60 | 2.60 | 26.20 | 1.00 | 69.00 | 1.75 | 67.00 | 13.00 | 238.00 | 9.25 | 495.00 | 11.40 |
| 14 | T0052 | 104.40 | 2.80 | 27.60 | 2.00 | 82.25 | 1.75 | 50.75 | 8.00 | 173.50 | 8.01 | 369.00 | 8.07 |
| 15 | T0061 | 98.80 | 2.80 | 23.60 | 1.50 | 144.50 | 2.25 | 124.75 | 9.00 | 195.00 | 8.87 | 476.50 | 29.26 |
| 16 | T0063 | 98.20 | 2.60 | 25.20 | 1.50 | 201.75 | 2.00 | 123.00 | 14.00 | 202.50 | 9.12 | 438.50 | 26.24 |
| 17 | T0066 | 99.40 | 3.00 | 23.80 | 1.25 | 193.50 | 2.25 | 160.00 | 10.00 | 214.25 | 10.88 | 653.00 | 28.31 |
| 18 | T0077 | 101.80 | 3.00 | 28.20 | 2.00 | 79.25 | 2.50 | 96.50 | 9.75 | 311.50 | 6.90 | 349.00 | 19.45 |
| 19 | T0082 | 114.40 | 3.60 | 29.40 | 2.00 | 247.00 | 2.00 | 165.00 | 16.75 | 315.50 | 9.34 | 786.00 | 26.12 |
| 20 | T0083 | 101.20 | 2.60 | 26.60 | 1.50 | 56.00 | 1.50 | 53.25 | 12.00 | 98.25 | 8.37 | 479.50 | 21.03 |
| 21 | T0084 | 98.80 | 2.60 | 24.40 | 1.50 | 137.50 | 2.75 | 145.25 | 11.50 | 189.75 | 7.88 | 616.50 | 24.74 |
| 22 | T0085 | 96.80 | 2.60 | 21.00 | 1.25 | 91.25 | 2.25 | 101.75 | 14.25 | 189.50 | 9.82 | 822.50 | 16.96 |
| 23 | T0093 | 93.60 | 3.20 | 27.20 | 1.25 | 97.50 | 2.25 | 91.75 | 12.25 | 144.50 | 12.00 | 516.00 | 18.97 |
| 24 | T0094 | 112.80 | 3.20 | 30.80 | 2.00 | 188.50 | 2.25 | 126.50 | 8.75 | 216.00 | 8.75 | 597.00 | 24.21 |
| 25 | T0095 | 103.40 | 3.00 | 25.20 | 1.25 | 55.25 | 2.00 | 49.00 | 16.00 | 217.75 | 9.17 | 458.50 | 18.25 |
| 26 | T0095-1 | 99.40 | 2.60 | 26.60 | 2.00 | 186.25 | 2.25 | 173.00 | 16.00 | 336.75 | 8.06 | 791.50 | 12.44 |
| 27 | T0096 | 99.48 | 3.41 | 10.41 | 1.00 | 140.13 | 1.00 | 71.31 | 6.25 | 141.87 | 10.14 | 351.82 | 28.47 |
| 28 | T0097 | 105.70 | 3.00 | 24.83 | 1.75 | 166.90 | 2.00 | 69.57 | 15.52 | 281.26 | 10.39 | 464.42 | 20.83 |
| 29 | T0098 | 99.69 | 3.00 | 27.83 | 1.00 | 111.35 | 1.75 | 101.09 | 6.75 | 151.89 | 10.89 | 192.67 | 13.31 |
| 30 | T0102 | 105.30 | 4.00 | 19.62 | 1.25 | 114.61 | 2.25 | 119.86 | 14.26 | 227.71 | 8.94 | 390.86 | 27.41 |
| 31 | T0103 | 99.09 | 3.00 | 27.43 | 1.51 | 151.89 | 2.51 | 239.96 | 11.01 | 170.91 | 8.76 | 558.51 | 28.89 |
| 32 | T0104 | 97.29 | 2.60 | 27.43 | 2.25 | 202.93 | 2.00 | 107.85 | 15.52 | 176.91 | 9.53 | 497.45 | 12.18 |
| 33 | T0105 | 101.29 | 2.81 | 27.63 | 1.51 | 152.14 | 1.75 | 97.09 | 16.52 | 266.74 | 9.07 | 563.01 | 19.62 |
| 34 | T0106 | 104.90 | 2.81 | 24.42 | 2.51 | 214.20 | 1.75 | 82.08 | 13.52 | 235.47 | 7.74 | 641.58 | 30.14 |
| 35 | T0107 | 93.40 | 3.00 | 23.60 | 1.51 | 74.00 | 8.75 | 105.25 | 11.00 | 32.25 | 7.05 | 203.00 | 16.00 |
| 36 | T0108 | 103.50 | 3.00 | 27.03 | 1.00 | 49.30 | 1.75 | 61.06 | 6.01 | 201.93 | 8.63 | 335.30 | 20.53 |
| 37 | T0109 | 107.50 | 3.00 | 32.23 | 1.00 | 44.79 | 1.51 | 43.79 | 9.01 | 112.85 | 8.63 | 151.13 | 7.02 |
| 38 | T0116 | 98.89 | 2.60 | 25.62 | 2.25 | 221.20 | 2.25 | 123.11 | 15.01 | 324.04 | 8.66 | 894.80 | 26.69 |
| 39 | T0117 | 109.10 | 3.21 | 29.43 | 3.00 | 305.03 | 2.76 | 114.61 | 12.76 | 272.50 | 10.60 | 591.03 | 30.85 |
| 40 | T0118 | 98.29 | 3.00 | 28.03 | 1.00 | 104.60 | 2.51 | 132.87 | 11.01 | 196.43 | 8.24 | 462.92 | 26.20 |
| 41 | T0119 | 108.25 | 3.60 | 28.61 | 1.26 | 109.04 | 1.50 | 66.03 | 9.76 | 196.33 | 8.44 | 350.14 | 29.35 |
| 42 | T0121 | 101.25 | 2.60 | 25.61 | 1.76 | 161.32 | 1.26 | 147.56 | 8.25 | 228.34 | 10.28 | 390.66 | 25.68 |
| 43 | T0122 | 98.04 | 3.21 | 22.81 | 1.00 | 46.02 | 2.00 | 43.27 | 13.51 | 195.83 | 7.50 | 394.66 | 16.28 |
| 44 | T0123 | 109.65 | 3.00 | 28.22 | 1.76 | 170.32 | 2.00 | 123.80 | 12.51 | 233.35 | 8.00 | 583.24 | 35.83 |
| 45 | T0124 | 100.04 | 2.81 | 24.01 | 1.00 | 132.81 | 2.00 | 53.02 | 9.50 | 173.57 | 12.51 | 490.70 | 17.47 |
| 46 | T0126 | 95.23 | 2.60 | 25.01 | 1.50 | 206.84 | 2.26 | 153.56 | 10.76 | 198.33 | 9.94 | 653.27 | 28.98 |
| 47 | T0127 | 61.80 | 2.20 | 17.00 | 1.75 | 36.51 | 3.25 | 46.00 | 7.25 | 23.75 | 5.44 | 191.00 | 4.50 |
| 48 | T0128 | 101.25 | 3.00 | 22.01 | 1.00 | 139.06 | 2.26 | 127.05 | 12.26 | 187.08 | 9.50 | 512.21 | 27.46 |
| 49 | T0129 | 107.25 | 3.00 | 25.41 | 2.50 | 206.84 | 2.26 | 130.81 | 12.00 | 306.88 | 8.22 | 784.32 | 29.51 |
| 50 | T0130 | 107.44 | 2.81 | 26.81 | 1.26 | 82.53 | 1.50 | 50.02 | 8.50 | 151.56 | 9.63 | 360.65 | 14.73 |
| 51 | T0132 | 100.24 | 2.81 | 26.41 | 1.00 | 99.54 | 1.50 | 65.78 | 11.26 | 210.09 | 8.31 | 457.19 | 17.07 |
| 52 | T0133 | 95.64 | 3.00 | 25.41 | 1.26 | 119.80 | 2.00 | 76.79 | 12.00 | 157.56 | 10.26 | 355.14 | 10.22 |
| 53 | T0134 | 99.44 | 3.00 | 30.22 | 1.00 | 114.05 | 1.00 | 60.02 | 9.76 | 110.04 | 10.46 | 304.12 | 11.91 |

PH= Plant Height; NB= Number of branches; NL= Number of leaves; NMR= Number of mother rhizome; WMR= Weight of mother rhizome; NPF= Number of primary fingers; WPF= Weight of primary finger; NSF= Number of secondary fingers; WSF= Weight of secondary finger; MRL= Length of mother rhizome; YPP= Yield per plant; FY= Fresh yield;
