# Supplementary material for: Study on the genetic variability and adaptability of turmeric (Curcuma longa L.) genotypes for development of desirable cultivars
Source: PLoS One. 2024 Jan 19;19(1):e0297202. doi: 10.1371/journal.pone.0297202 (PMC10798502; doi:10.1371/journal.pone.0297202)
Supplement: S8 Table — (DOCX) [file pone.0297202.s008.docx]

**Table S8.** Mean performance of 53 genotypes of turmeric grown during the year of 2021-22

| **Sl. No.** | **Genotype** | **PH** | **NB** | **NL** | **NMR** | **WMR** | **NPF** | **WPF** | **NSF** | **WSF** | **LMR** | **YPP** | **FY** |
| --- | --- | --- | --- | --- | --- | --- | --- | --- | --- | --- | --- | --- | --- |
| 1 | BARI Holud-1 | 89.69 | 5.60 | 20.80 | 1.50 | 32.26 | 7.00 | 191.50 | 9.00 | 185.00 | 6.95 | 408.75 | 17.00 |
| 2 | BARI Holud-2 | 107.40 | 5.00 | 26.00 | 1.76 | 84.00 | 6.26 | 133.76 | 9.25 | 101.00 | 7.58 | 318.76 | 12.60 |
| 3 | BARI Holud-3 | 117.60 | 5.60 | 24.00 | 1.50 | 109.50 | 6.76 | 121.50 | 8.50 | 107.50 | 6.93 | 338.50 | 35.00 |
| 4 | BARI Holud-4 | 104.40 | 6.00 | 22.80 | 1.50 | 130.75 | 6.00 | 164.26 | 12.26 | 158.00 | 7.20 | 453.00 | 39.30 |
| 5 | BARI Holud-5 | 124.20 | 7.40 | 30.00 | 1.00 | 52.25 | 5.50 | 103.76 | 5.76 | 41.26 | 7.65 | 198.26 | 24.00 |
| 6 | T0008 | 127.60 | 5.00 | 25.60 | 1.26 | 109.25 | 7.26 | 132.50 | 9.50 | 97.76 | 8.02 | 322.00 | 21.70 |
| 7 | T0012 | 89.40 | 5.20 | 23.00 | 1.76 | 65.25 | 7.00 | 123.50 | 11.00 | 74.26 | 8.05 | 263.00 | 11.00 |
| 8 | T0013 | 97.00 | 8.40 | 28.20 | 1.50 | 102.50 | 6.50 | 133.00 | 8.75 | 78.76 | 7.70 | 314.26 | 6.30 |
| 9 | T0015 | 108.20 | 7.80 | 26.60 | 1.00 | 99.25 | 5.26 | 154.76 | 6.00 | 79.50 | 8.80 | 314.76 | 45.00 |
| 10 | T0016 | 89.20 | 4.40 | 18.60 | 1.26 | 53.26 | 3.76 | 70.00 | 12.50 | 204.76 | 6.88 | 325.76 | 17.50 |
| 11 | T0017 | 125.20 | 6.60 | 35.60 | 1.00 | 35.00 | 7.25 | 76.25 | 10.25 | 91.25 | 7.13 | 205.00 | 10.00 |
| 12 | T0019 | 92.40 | 7.20 | 26.00 | 1.00 | 40.00 | 7.51 | 65.75 | 6.25 | 59.25 | 5.42 | 165.50 | 14.00 |
| 13 | T0023 | 100.40 | 2.60 | 27.40 | 1.25 | 130.51 | 1.25 | 49.75 | 8.00 | 109.75 | 9.13 | 514.51 | 15.82 |
| 14 | T0052 | 86.60 | 4.20 | 18.20 | 1.25 | 48.25 | 3.00 | 59.00 | 4.50 | 28.75 | 6.25 | 136.00 | 9.50 |
| 15 | T0061 | 112.20 | 5.40 | 25.00 | 1.25 | 133.00 | 7.00 | 141.75 | 5.75 | 62.75 | 8.55 | 336.00 | 41.00 |
| 16 | T0063 | 113.60 | 10.20 | 31.80 | 1.00 | 68.75 | 7.25 | 99.00 | 6.25 | 57.75 | 7.60 | 225.51 | 14.00 |
| 17 | T0066 | 93.00 | 4.20 | 21.60 | 1.25 | 71.00 | 9.75 | 135.51 | 7.00 | 87.00 | 5.73 | 293.75 | 26.00 |
| 18 | T0077 | 86.00 | 5.60 | 17.20 | 2.75 | 84.75 | 5.51 | 179.25 | 7.25 | 140.00 | 5.35 | 404.00 | 11.00 |
| 19 | T0082 | 105.40 | 8.20 | 32.80 | 1.75 | 110.00 | 6.00 | 104.75 | 9.00 | 69.75 | 9.05 | 284.51 | 27.20 |
| 20 | T0083 | 112.60 | 8.00 | 35.20 | 1.51 | 101.00 | 6.51 | 89.25 | 6.75 | 82.25 | 7.35 | 272.51 | 30.00 |
| 21 | T0084 | 114.20 | 5.00 | 19.80 | 2.00 | 74.00 | 6.50 | 79.26 | 8.76 | 105.00 | 7.35 | 258.26 | 20.70 |
| 22 | T0085 | 97.60 | 5.80 | 27.20 | 1.26 | 67.26 | 6.00 | 71.00 | 6.50 | 55.00 | 5.98 | 193.25 | 25.00 |
| 23 | T0093 | 100.40 | 4.20 | 28.80 | 1.00 | 112.76 | 8.50 | 166.00 | 11.00 | 150.50 | 8.83 | 429.26 | 24.50 |
| 24 | T0094 | 110.20 | 4.20 | 22.80 | 2.00 | 47.76 | 6.76 | 55.75 | 7.25 | 65.75 | 7.08 | 169.26 | 28.00 |
| 25 | T0095 | 94.80 | 4.40 | 28.00 | 1.50 | 25.50 | 9.26 | 45.26 | 6.76 | 28.50 | 7.15 | 99.26 | 6.00 |
| 26 | T0095-1 | 117.80 | 7.00 | 21.80 | 1.00 | 73.26 | 5.00 | 92.50 | 4.75 | 34.00 | 7.95 | 199.76 | 28.00 |
| 27 | T0096 | 121.40 | 4.40 | 25.60 | 1.00 | 60.26 | 5.26 | 76.50 | 7.00 | 36.25 | 8.38 | 173.00 | 21.50 |
| 28 | T0097 | 118.20 | 4.20 | 24.40 | 1.50 | 120.00 | 8.26 | 183.00 | 8.26 | 104.26 | 7.63 | 407.26 | 27.30 |
| 29 | T0098 | 109.60 | 4.60 | 22.69 | 1.50 | 97.76 | 9.76 | 115.76 | 8.25 | 65.26 | 6.80 | 288.76 | 13.50 |
| 30 | T0102 | 123.00 | 6.00 | 24.80 | 1.26 | 58.50 | 6.76 | 65.50 | 6.75 | 55.00 | 6.76 | 179.00 | 27.30 |
| 31 | T0103 | 113.40 | 10.80 | 36.60 | 1.00 | 25.25 | 4.75 | 116.25 | 4.75 | 57.25 | 7.88 | 198.75 | 35.00 |
| 32 | T0104 | 94.60 | 4.40 | 20.80 | 1.00 | 37.25 | 4.00 | 70.75 | 7.50 | 46.00 | 8.20 | 154.00 | 9.50 |
| 33 | T0105 | 101.60 | 5.00 | 23.80 | 1.25 | 51.25 | 4.51 | 68.75 | 5.00 | 46.51 | 7.55 | 166.51 | 7.40 |
| 34 | T0106 | 107.80 | 7.60 | 35.40 | 1.25 | 61.51 | 7.00 | 93.00 | 7.25 | 106.25 | 6.68 | 218.25 | 16.60 |
| 35 | T0107 | 95.51 | 5.20 | 29.60 | 1.75 | 112.51 | 5.00 | 138.75 | 11.25 | 106.51 | 8.83 | 357.75 | 18.30 |
| 36 | T0108 | 83.00 | 4.40 | 14.00 | 1.25 | 60.75 | 7.25 | 195.25 | 6.75 | 77.00 | 7.98 | 321.75 | 18.70 |
| 37 | T0109 | 105.20 | 4.20 | 15.00 | 1.25 | 40.00 | 3.25 | 47.50 | 3.75 | 38.25 | 6.28 | 161.25 | 4.80 |
| 38 | T0116 | 115.40 | 7.00 | 28.80 | 1.25 | 42.00 | 5.25 | 63.75 | 5.51 | 57.75 | 6.33 | 163.51 | 26.00 |
| 39 | T0117 | 116.40 | 6.60 | 23.80 | 1.25 | 49.00 | 5.00 | 121.75 | 6.00 | 57.51 | 7.05 | 228.25 | 23.00 |
| 40 | T0118 | 130.40 | 5.60 | 29.40 | 1.25 | 87.25 | 4.00 | 196.00 | 5.75 | 160.00 | 6.95 | 443.25 | 23.00 |
| 41 | T0119 | 111.40 | 5.40 | 24.60 | 2.00 | 91.75 | 7.25 | 148.25 | 8.75 | 57.75 | 8.60 | 286.51 | 11.00 |
| 42 | T0121 | 113.80 | 5.00 | 25.40 | 3.00 | 144.75 | 11.00 | 123.75 | 13.51 | 113.51 | 7.00 | 382.00 | 24.20 |
| 43 | T0122 | 88.00 | 3.60 | 21.00 | 1.25 | 37.00 | 6.00 | 104.00 | 7.75 | 73.00 | 6.05 | 214.00 | 14.00 |
| 44 | T0123 | 119.80 | 4.20 | 22.20 | 1.00 | 68.75 | 7.00 | 42.75 | 6.25 | 60.51 | 7.73 | 169.51 | 11.91 |
| 45 | T0124 | 127.80 | 6.00 | 23.00 | 1.51 | 97.00 | 6.51 | 101.51 | 6.00 | 59.25 | 8.68 | 257.75 | 9.30 |
| 46 | T0126 | 89.60 | 6.20 | 23.80 | 1.00 | 82.51 | 4.00 | 187.00 | 15.25 | 366.00 | 8.15 | 635.51 | 20.80 |
| 47 | T0127 | 86.60 | 5.60 | 20.60 | 1.51 | 31.51 | 5.51 | 72.50 | 6.25 | 80.00 | 6.80 | 184.00 | 4.00 |
| 48 | T0128 | 110.91 | 6.11 | 24.40 | 1.51 | 56.25 | 5.25 | 69.50 | 8.75 | 60.75 | 7.05 | 186.51 | 10.51 |
| 49 | T0129 | 121.20 | 4.80 | 24.60 | 1.00 | 59.25 | 6.00 | 117.75 | 8.75 | 113.75 | 6.00 | 290.75 | 27.00 |
| 50 | T0130 | 91.00 | 3.80 | 19.60 | 2.00 | 40.25 | 4.25 | 47.25 | 6.50 | 38.50 | 5.93 | 126.00 | 8.10 |
| 51 | T0132 | 112.40 | 5.00 | 29.20 | 3.25 | 300.00 | 15.25 | 304.75 | 13.25 | 82.00 | 9.45 | 686.75 | 23.31 |
| 52 | T0133 | 103.60 | 4.20 | 18.80 | 1.00 | 24.25 | 5.00 | 71.50 | 6.50 | 41.00 | 5.98 | 136.75 | 21.00 |
| 53 | T0134 | 97.60 | 5.00 | 19.20 | 1.75 | 108.25 | 9.25 | 201.00 | 13.00 | 139.00 | 7.51 | 449.25 | 18.51 |

PH= Plant Height; NB= Number of branches; NL= Number of leaves; NMR= Number of mother rhizome; WMR= Weight of mother rhizome; NPF= Number of primary fingers; WPF= Weight of primary finger; NSF= Number of secondary fingers; WSF= Weight of secondary finger; MRL= Length of mother rhizome; YPP= Yield per plant; FY= Fresh yield;
