# Supplementary material for: Study on the genetic variability and adaptability of turmeric (Curcuma longa L.) genotypes for development of desirable cultivars
Source: PLoS One. 2024 Jan 19;19(1):e0297202. doi: 10.1371/journal.pone.0297202 (PMC10798502; doi:10.1371/journal.pone.0297202)
Supplement: S10 Table — (DOCX) [file pone.0297202.s010.docx]

Table S10. Association of studied traits based on the performances during the year of 2020-21

|  | Type | PH | NB | NL | NMR | WMR | NPF | WPF | NSF | WSF | LMR | YPP |
| --- | --- | --- | --- | --- | --- | --- | --- | --- | --- | --- | --- | --- |
| NB | r_g_ | 0.33^*^ |  |  |  |  |  |  |  |  |  |  |
|  | r_p_ | 0.44^**^ |  |  |  |  |  |  |  |  |  |  |
| NL | r_g_ | 0.37^**^ | -0.03 |  |  |  |  |  |  |  |  |  |
|  | r_p_ | 0.44^**^ | 0.05 |  |  |  |  |  |  |  |  |  |
| NMR | r_g_ | 0.13 | -0.23 | 0.09 |  |  |  |  |  |  |  |  |
|  | r_p_ | 0.17 | -0.16 | 0.12 |  |  |  |  |  |  |  |  |
| WMR | r_g_ | 0.31^*^ | 0.10 | 0.05 | 0.62^**^ |  |  |  |  |  |  |  |
|  | r_p_ | 0.30^*^ | 0.11 | 0.07 | 0.63^**^ |  |  |  |  |  |  |  |
| NPF | r_g_ | 0.38^**^ | -0.04 | -0.14 | 0.13 | -0.01 |  |  |  |  |  |  |
|  | r_p_ | 0.28^*^ | -0.02 | -0.11 | 0.14 | -0.01 |  |  |  |  |  |  |
| WPF | r_g_ | 0.11 | -0.01 | -0.01 | 0.30^*^ | 0.63^**^ | 0.22 |  |  |  |  |  |
|  | r_p_ | 0.14 | 0.01 | 0.01 | 0.31^*^ | 0.63^**^ | 0.22 |  |  |  |  |  |
| NSF | r_g_ | 0.05 | -0.07 | 0.00 | 0.25 | 0.40^**^ | 0.11 | 0.33^*^ |  |  |  |  |
|  | r_p_ | 0.12 | -0.01 | 0.04 | 0.26 | 0.40^**^ | 0.13 | 0.34^*^ |  |  |  |  |
| WSF | r_g_ | 0.40^**^ | -0.14 | 0.06 | 0.39^**^ | 0.44^**^ | -0.20 | 0.39^**^ | 0.52^**^ |  |  |  |
|  | r_p_ | 0.46^**^ | 0.01 | 0.13 | 0.41^**^ | 0.53^**^ | -0.18 | 0.47^**^ | 0.58^**^ |  |  |  |
| LMR | r_g_ | 0.09 | 0.18 | 0.02 | -0.30^*^ | 0.31^*^ | -0.34^*^ | 0.03 | -0.04 | -0.01 |  |  |
|  | r_p_ | 0.21 | 0.25 | 0.08 | -0.25 | 0.32^*^ | -0.30^*^ | 0.06 | 0.00 | 0.02 |  |  |
| YPP | r_g_ | 0.14 | -0.14 | -0.08 | 0.42^**^ | 0.64^**^ | 0.01 | 0.60^**^ | 0.69^**^ | 0.60^**^ | 0.07 |  |
|  | r_p_ | 0.17 | -0.10 | -0.05 | 0.42^**^ | 0.65^**^ | 0.02 | 0.61^**^ | 0.69^**^ | 0.67^**^ | 0.10 |  |
| FY | r_g_ | 0.32^*^ | 0.38^**^ | -0.04 | 0.19 | 0.55^**^ | -0.03 | 0.44^**^ | 0.07 | 0.24 | 0.15 | 0.37^**^ |
|  | r_p_ | 0.33^*^ | 0.43^**^ | -0.01 | 0.19 | 0.56^**^ | -0.02 | 0.45^**^ | 0.08 | 0.22 | 0.17 | 0.37^**^ |

PH= Plant Height; NB= Number of branches; NL= Number of leaves; NMR= Number of mother rhizome; WMR= Weight of mother rhizome; NPF= Number of primary fingers; WPF= Weight of primary finger; NSF= Number of secondary fingers; WSF= Weight of secondary finger; MRL= Length of mother rhizome; YPP= Yield per plant; FY= Fresh yield; r_g_=Genotypic correlation; r_p_=Phenotypic correlation;
