# Supplementary material for: Study on the genetic variability and adaptability of turmeric (Curcuma longa L.) genotypes for development of desirable cultivars
Source: PLoS One. 2024 Jan 19;19(1):e0297202. doi: 10.1371/journal.pone.0297202 (PMC10798502; doi:10.1371/journal.pone.0297202)
Supplement: S12 Table — (DOCX) [file pone.0297202.s012.docx]

**Table S12.** Geometric adaptability index of the studied genotypes evaluated over three consecutive years

| **Sl. No.** | **Genotypes** | **PH** | **NB** | **NL** | **NMR** | **WMR** | **NPF** | **WPF** | **NSF** | **WSF** | **LMR** | **YPP** | **FY** |
| --- | --- | --- | --- | --- | --- | --- | --- | --- | --- | --- | --- | --- | --- |
| 1 | BARI Holud-1 | 90.82 | 3.00 | 19.96 | 1.87 | 49.72 | 4.07 | 93.03 | 8.46 | 128.19 | 6.71 | 311.07 | 16.36 |
| 2 | BARI Holud-2 | 89.40 | 3.00 | 21.73 | 1.40 | 56.27 | 3.83 | 63.76 | 8.66 | 76.97 | 7.44 | 188.11 | 10.63 |
| 3 | BARI Holud-3 | 97.40 | 3.26 | 20.50 | 1.74 | 85.69 | 3.62 | 84.02 | 8.64 | 76.36 | 6.75 | 258.54 | 15.84 |
| 4 | BARI Holud-4 | 105.07 | 3.43 | 22.90 | 1.49 | 96.84 | 4.29 | 101.26 | 10.50 | 103.36 | 8.10 | 318.86 | 19.53 |
| 5 | BARI Holud-5 | 102.19 | 3.46 | 24.97 | 1.45 | 53.40 | 3.70 | 72.67 | 8.41 | 57.37 | 6.61 | 236.99 | 15.41 |
| 6 | T0008 | 99.33 | 3.06 | 20.24 | 1.30 | 96.93 | 4.39 | 100.26 | 10.35 | 100.33 | 7.63 | 317.70 | 11.89 |
| 7 | T0012 | 89.48 | 3.00 | 19.86 | 1.58 | 73.51 | 4.58 | 100.38 | 11.52 | 76.54 | 6.81 | 284.04 | 10.85 |
| 8 | T0013 | 82.37 | 3.35 | 20.38 | 1.38 | 51.74 | 4.20 | 81.32 | 10.74 | 71.92 | 7.53 | 230.32 | 7.02 |
| 9 | T0015 | 101.76 | 3.93 | 24.05 | 1.30 | 86.33 | 3.97 | 83.63 | 8.61 | 71.08 | 7.94 | 266.19 | 23.77 |
| 10 | T0016 | 90.93 | 3.02 | 22.96 | 1.76 | 53.82 | 3.98 | 76.14 | 11.38 | 134.52 | 6.16 | 348.22 | 14.65 |
| 11 | T0017 | 101.36 | 3.29 | 26.08 | 1.00 | 57.64 | 4.12 | 71.54 | 10.51 | 92.08 | 8.45 | 226.23 | 12.51 |
| 12 | T0019 | 86.29 | 4.19 | 24.55 | 1.46 | 82.36 | 5.74 | 89.75 | 11.19 | 60.25 | 6.73 | 332.93 | 20.51 |
| 13 | T0023 | 96.30 | 2.38 | 23.29 | 1.16 | 74.00 | 2.51 | 68.87 | 10.13 | 109.13 | 8.00 | 388.36 | 13.02 |
| 14 | T0052 | 82.01 | 2.54 | 19.78 | 1.55 | 50.00 | 2.43 | 47.92 | 4.76 | 40.18 | 5.75 | 157.60 | 5.56 |
| 15 | T0061 | 103.29 | 3.57 | 21.98 | 1.49 | 114.08 | 4.42 | 105.81 | 7.68 | 67.60 | 7.65 | 351.39 | 22.97 |
| 16 | T0063 | 100.50 | 3.88 | 23.31 | 1.14 | 78.78 | 4.03 | 80.29 | 7.72 | 60.57 | 7.67 | 247.33 | 12.49 |
| 17 | T0066 | 97.61 | 3.43 | 20.76 | 1.33 | 92.71 | 4.25 | 99.00 | 8.73 | 87.75 | 6.67 | 379.08 | 19.58 |
| 18 | T0077 | 79.41 | 3.12 | 17.47 | 1.90 | 44.93 | 3.80 | 85.20 | 5.96 | 77.08 | 5.10 | 262.30 | 6.85 |
| 19 | T0082 | 106.93 | 4.25 | 25.99 | 1.74 | 129.13 | 3.98 | 107.06 | 13.20 | 104.93 | 8.49 | 443.08 | 24.38 |
| 20 | T0083 | 94.81 | 3.78 | 26.01 | 1.41 | 57.58 | 3.65 | 49.65 | 8.07 | 53.78 | 6.85 | 222.23 | 10.62 |
| 21 | T0084 | 102.91 | 3.54 | 24.22 | 1.82 | 76.36 | 4.88 | 98.19 | 9.30 | 90.13 | 7.60 | 269.54 | 20.16 |
| 22 | T0085 | 101.66 | 3.64 | 25.31 | 1.52 | 101.67 | 4.66 | 104.51 | 9.99 | 98.79 | 7.88 | 429.65 | 21.97 |
| 23 | T0093 | 93.79 | 3.84 | 28.32 | 1.08 | 74.92 | 4.99 | 94.27 | 8.91 | 61.12 | 8.73 | 356.18 | 14.82 |
| 24 | T0094 | 108.16 | 3.64 | 26.20 | 2.15 | 96.15 | 5.10 | 82.89 | 8.52 | 79.03 | 7.37 | 314.21 | 25.79 |
| 25 | T0095 | 93.73 | 3.33 | 24.25 | 1.49 | 40.74 | 4.60 | 52.16 | 9.52 | 72.24 | 6.77 | 222.18 | 9.32 |
| 26 | T0095-1 | 95.59 | 3.42 | 19.90 | 1.36 | 69.40 | 3.40 | 70.07 | 6.58 | 51.60 | 6.81 | 260.65 | 9.01 |
| 27 | T0096 | 105.06 | 3.39 | 17.29 | 1.15 | 93.24 | 3.78 | 70.05 | 6.98 | 73.28 | 8.19 | 235.28 | 18.41 |
| 28 | T0097 | 105.80 | 3.28 | 22.81 | 1.58 | 99.38 | 4.50 | 87.57 | 9.97 | 85.74 | 7.44 | 343.34 | 16.22 |
| 29 | T0098 | 101.04 | 3.38 | 21.80 | 1.38 | 76.73 | 4.98 | 91.40 | 8.56 | 70.60 | 7.07 | 266.18 | 12.01 |
| 30 | T0102 | 101.53 | 4.16 | 20.22 | 1.40 | 77.34 | 4.37 | 78.13 | 8.97 | 81.04 | 7.39 | 255.36 | 19.43 |
| 31 | T0103 | 112.76 | 4.89 | 30.55 | 1.44 | 81.57 | 4.61 | 162.56 | 7.92 | 85.66 | 7.94 | 391.84 | 26.30 |
| 32 | T0104 | 88.06 | 3.10 | 20.36 | 1.31 | 52.14 | 3.04 | 59.60 | 7.04 | 43.33 | 7.91 | 227.65 | 6.34 |
| 33 | T0105 | 94.58 | 3.14 | 22.00 | 1.55 | 86.06 | 4.14 | 77.32 | 9.22 | 106.99 | 7.64 | 335.82 | 12.10 |
| 34 | T0106 | 109.28 | 4.08 | 28.08 | 1.76 | 112.64 | 4.51 | 94.20 | 10.01 | 118.94 | 7.42 | 460.63 | 24.10 |
| 35 | T0107 | 98.33 | 3.52 | 25.23 | 1.38 | 77.77 | 5.47 | 86.62 | 11.87 | 47.27 | 7.61 | 181.26 | 15.03 |
| 36 | T0108 | 79.91 | 3.07 | 17.99 | 1.36 | 53.80 | 4.71 | 118.90 | 6.65 | 91.80 | 7.53 | 334.52 | 17.39 |
| 37 | T0109 | 89.41 | 2.93 | 18.26 | 1.08 | 34.16 | 2.38 | 36.56 | 5.13 | 41.87 | 5.94 | 106.09 | 3.78 |
| 38 | T0116 | 100.67 | 3.88 | 26.14 | 1.41 | 77.70 | 3.62 | 60.88 | 7.80 | 70.84 | 7.18 | 311.20 | 17.10 |
| 39 | T0117 | 116.34 | 3.70 | 24.50 | 1.87 | 114.24 | 4.64 | 110.42 | 10.35 | 105.41 | 8.45 | 384.39 | 26.44 |
| 40 | T0118 | 105.19 | 3.52 | 24.75 | 1.16 | 68.36 | 3.27 | 99.65 | 8.05 | 102.73 | 6.87 | 290.92 | 18.20 |
| 41 | T0119 | 105.36 | 3.50 | 22.60 | 1.64 | 92.36 | 3.91 | 84.21 | 9.07 | 69.22 | 7.82 | 252.25 | 17.39 |
| 42 | T0121 | 110.47 | 3.39 | 26.12 | 2.19 | 142.05 | 5.07 | 139.99 | 11.53 | 140.93 | 7.38 | 601.46 | 23.91 |
| 43 | T0122 | 80.02 | 3.33 | 20.35 | 1.08 | 25.51 | 2.88 | 43.66 | 6.98 | 50.95 | 5.71 | 241.13 | 8.06 |
| 44 | T0123 | 102.67 | 2.83 | 20.01 | 1.52 | 103.25 | 4.25 | 58.46 | 7.77 | 62.75 | 7.52 | 306.06 | 14.80 |
| 45 | T0124 | 96.55 | 3.23 | 19.19 | 1.23 | 80.50 | 4.27 | 71.00 | 7.53 | 68.50 | 8.38 | 210.74 | 8.90 |
| 46 | T0126 | 89.14 | 3.18 | 20.56 | 1.38 | 101.48 | 3.50 | 110.90 | 12.08 | 125.91 | 7.42 | 355.26 | 11.56 |
| 47 | T0127 | 70.33 | 3.00 | 17.46 | 1.38 | 25.69 | 3.77 | 39.16 | 5.77 | 29.12 | 5.99 | 134.35 | 3.98 |
| 48 | T0128 | 94.43 | 3.32 | 19.31 | 1.44 | 76.39 | 4.14 | 72.41 | 9.88 | 73.72 | 7.09 | 214.97 | 8.72 |
| 49 | T0129 | 116.10 | 3.43 | 25.20 | 1.55 | 95.36 | 4.27 | 105.04 | 9.44 | 109.39 | 7.06 | 397.78 | 25.47 |
| 50 | T0130 | 87.70 | 2.46 | 18.16 | 1.46 | 42.28 | 2.75 | 39.96 | 6.51 | 43.90 | 6.88 | 156.90 | 7.18 |
| 51 | T0132 | 99.11 | 3.55 | 26.96 | 1.60 | 123.29 | 4.51 | 123.07 | 11.97 | 116.49 | 7.93 | 434.91 | 16.19 |
| 52 | T0133 | 92.93 | 3.12 | 19.53 | 1.36 | 51.86 | 3.92 | 61.44 | 7.18 | 50.76 | 7.24 | 187.16 | 10.71 |
| 53 | T0134 | 91.83 | 3.63 | 23.79 | 1.64 | 100.82 | 3.47 | 95.75 | 10.91 | 104.22 | 7.95 | 409.06 | 10.54 |

PH= Plant Height; NB= Number of branches; NL= Number of leaves; NMR= Number of mother rhizome; WMR= Weight of mother rhizome; NPF= Number of primary fingers; WPF= Weight of primary finger; NSF= Number of secondary fingers; WSF= Weight of secondary finger; MRL= Length of mother rhizome; YPP= Yield per plant; FY= Fresh yield;
